# GPR43 stimulation on TCRαβ^+^ intraepithelial colonic lymphocytes inhibits the recruitment of encephalitogenic T-cells into the central nervous system and attenuates the development of autoimmunity

**DOI:** 10.1186/s12974-023-02815-9

**Published:** 2023-06-01

**Authors:** Carolina Prado, Alexandra Espinoza, J. Eduardo Martínez-Hernández, Joseph Petrosino, Erick Riquelme, Alberto J. M. Martin, Rodrigo Pacheco

**Affiliations:** 1 Laboratorio de Neuroinmunología, Centro Científico y Tecnológico de Excelencia Ciencia & Vida, Fundación Ciencia & Vida, Avenida Del Valle Norte #725, 8580702 Huechuraba, Santiago Chile; 2 Facultad de Medicina y Ciencia, Universidad San Sebastián, 7510156 Providencia, Santiago Chile; 3 Laboratorio de Redes Biológicas, Centro Científico y Tecnológico de Excelencia Ciencia & Vida, Fundación Ciencia & Vida, Avenida Del Valle Norte #725, 8580702 Huechuraba, Santiago Chile; 4 Department of Molecular Virology and Microbiology, Baylor College of Medicine, Houston, TX USA; 5 Respiratory Diseases Department, Faculty of Medicine, Pontifical Catholic University of Chile, Santiago, Chile; 6 Escuela de Ingeniería, Facultad de Ingeniería Arquitectura y Diseño, Universidad San Sebastián, Providencia, Chile; 7 Agriaquaculture Nutritional Genomic Center, Temuco, Chile

**Keywords:** Short-chain fatty acids, GPR43, Mucosal immunity, Gut-brain axis, Neuroinflammation, Experimental autoimmune encephalomyelitis

## Abstract

**Introduction:**

Gut microbiota plays a critical role in the regulation of immune homeostasis. Accordingly, several autoimmune disorders have been associated with dysbiosis in the gut microbiota. Notably, the dysbiosis associated with central nervous system (CNS) autoimmunity involves a substantial reduction of bacteria belonging to *Clostridia* clusters IV and XIVa, which constitute major producers of short-chain fatty acids (SCFAs). Here we addressed the role of the surface receptor-mediated effects of SCFAs on mucosal T-cells in the development of CNS autoimmunity.

**Methods:**

To induce CNS autoimmunity, we used the mouse model of experimental autoimmune encephalomyelitis (EAE) induced by immunization with the myelin oligodendrocyte glycoprotein (MOG)-derived peptide (MOG_35-55_ peptide). To address the effects of GPR43 stimulation on colonic TCRαβ^+^ T-cells upon CNS autoimmunity, mucosal lymphocytes were isolated and stimulated with a selective GPR43 agonist ex vivo and then transferred into congenic mice undergoing EAE. Several subsets of lymphocytes infiltrating the CNS or those present in the gut epithelium and gut lamina propria were analysed by flow cytometry. In vitro migration assays were conducted with mucosal T-cells using transwells.

**Results:**

Our results show a sharp and selective reduction of intestinal propionate at the peak of EAE development, accompanied by increased IFN-γ and decreased IL-22 in the colonic mucosa. Further analyses indicated that GPR43 was the primary SCFAs receptor expressed on T-cells, which was downregulated on colonic TCRαβ^+^ T-cells upon CNS autoimmunity. The pharmacologic stimulation of GPR43 increased the anti-inflammatory function and reduced the pro-inflammatory features in several TCRαβ^+^ T-cell subsets in the colonic mucosa upon EAE development. Furthermore, GPR43 stimulation induced the arrest of CNS-autoreactive T-cells in the colonic lamina propria, thus avoiding their infiltration into the CNS and dampening the disease development. Mechanistic analyses revealed that GPR43-stimulation on mucosal TCRαβ^+^ T-cells inhibits their CXCR3-mediated migration towards CXCL11, which is released from the CNS upon neuroinflammation.

**Conclusions:**

These findings provide a novel mechanism involved in the gut-brain axis by which bacterial-derived products secreted in the gut mucosa might control the CNS tropism of autoreactive T-cells. Moreover, this study shows GPR43 expressed on T-cells as a promising therapeutic target for CNS autoimmunity.

**Supplementary Information:**

The online version contains supplementary material available at 10.1186/s12974-023-02815-9.

## Introduction

Host organisms and commensal microbiota have evolved to establish a symbiotic relationship by secreting and sensing many common mediators [1]. Thus, commensal microbiota in the gastrointestinal tract might produce neurotransmitters, metabolites, and fatty acids that affect several host processes, including neural circuits, metabolism, behaviour, hormone secretion, and the immune response [1, 2]. According to the beneficial influence of gut microbiota in host homeostasis, significant alterations in the composition of this bacterial consortium involving reduced representation of beneficial bacteria or increased representation of pathogenic bacteria (dysbiosis) and derived products have been extensively associated with the pathogenesis of immune-related disorders, including autoimmune diseases [3–6].

Multiple sclerosis (MS) involves an autoimmune response to antigens derived from the central nervous system (CNS), which produces neuroinflammation and demyelination, promoting a broad spectrum of symptoms, including motor impairment, cognitive issues, and alterations in pain and itching perception [7]. The CNS autoimmunity in MS is driven by autoreactive T-cells bearing inflammatory phenotypes, such as T-helper 1 (Th1) and Th17 [7]. Nevertheless, many other immune actors play significant roles in this CNS autoimmunity, including local components such as microglia and astrocytes [8–10] and peripheral immune cells infiltrating the CNS, including B-cells [11], monocytes/macrophages [12] and neutrophils [13] among others. Notably, a previous study performed with monozygotic twin pairs discordant for MS demonstrated that the disease development relies on differences in the composition of the gut microbiota [14]. According to the critical role of the commensal microbiota in the development of CNS autoimmunity, experimental autoimmune encephalomyelitis (EAE) was strongly attenuated in mice when bred in germ-free conditions [15]. Of note, this reduction in EAE manifestation was accompanied by decreased levels of Th1 and Th17 in the CNS and the intestine and a reciprocal increase in the levels of regulatory T-cells (Treg), a subset of T-cells with immunosuppressive function [15].

A significant group of mediators produced by some bacteria of the intestinal microbiota corresponds to the short-chain fatty acids (SCFAs), which are products of bacterial fermentation and include acetate, propionate, and butyrate, among others. SCFAs can act on T-cell physiology either by altering the activity of epigenetic enzymes that regulate the transcriptional profile of these cells or through the stimulation of surface receptors [16]. Regarding the former mechanism, some SCFAs might inhibit the activity of histone deacetylase and consequently modify the epigenetic landscape of the chromatin in T-cells [17]. For instance, it has been described that propionate and butyrate enhance the degree of acetylation of the *foxp3* locus, inducing a more robust Foxp3 expression and, thereby, a substantial increase of Treg differentiation and higher suppressive activity [18]. In the same direction, another study shows that through butyrate production and the consequent inhibition of histone deacetylases, *Clostridium butyricum* B1 promotes the differentiation of T-cells into Th22 [19]. This subset of CD4^+^ T-cells is characterized by the secretion of IL-22, which increases the expression of the tight junctions on epithelial cells of the intestinal mucosa, thereby enhancing the barrier function [20]. Regarding the SCFAs effects mediated by the stimulation of cell surface receptors, an increasing body of studies has shown GPR41, GPR43, and GPR109A as the primary SCFAs receptors expressed on immune cells. For instance, the propionate- or butyrate-mediated GPR41 stimulation has been described to impair Th2 responses. Thus, GPR41-signalling on T-cells induces a protective effect in the airways in the context of allergic inflammation [21]. Moreover, GPR43 stimulation induced by acetate or propionate has been shown to promote the expansion and enhance the suppressive activity of colonic Treg, thereby dampening gut inflammation [22]. Similarly, the stimulation of GPR109A on colonic macrophages and dendritic cells mediated by niacin or butyrate exerts anti-inflammatory effects on these cells, which consequently induce a higher suppressive activity on Treg and inhibit the pro-inflammatory function of Th17 cells [23]. Altogether these works indicate that SCFAs induce anti-inflammatory effects in mucosal immunity by promoting Treg activity and increasing barrier function.

Interestingly, the dysbiosis associated with MS involves a substantial reduction of bacteria belonging to *Clostridia* clusters IV and XIVa, which constitute major producers of SCFAs [24]. Furthermore, recent evidence has shown that oral administration of SCFAs reduces the severity of EAE manifestation [25–27]. Thereby, the current evidence suggests that SCFAs exert anti-inflammatory effects being beneficial in the context of CNS autoimmunity. In this regard, a recent study addressed the role of GPR41- and GPR43-mediated effects on EAE using *gpr41* and *gpr43* knockout mice, respectively. Unexpectedly, the genetic deficiency of these *gpr41* or *gpr43* resulted in decreased disease severity [27], suggesting that SCFAs effects on EAE involve a complex regulation of the immune response through multiple cell types and multiple molecular targets.

Here we addressed the role of the surface receptor-mediated effects of SCFAs on T-cells in the development of CNS autoimmunity. Our results indicate that GPR43 was the primary receptor expressed on T-cells. Its stimulation increased anti-inflammatory function and reduced pro-inflammatory features in several T-cell subsets in the colonic mucosa upon EAE development. Furthermore, GPR43 stimulation induced the arrest of CNS-autoreactive T-cells in the colonic mucosa, thus avoiding their infiltration into the CNS and dampening the disease development.

## Materials and methods

### Animals

Six- to eight-week-old male or female mice were used in all experiments. Wild-type (WT) *Cd45.1*^+*/*+^, *Cd45.1*^±^ *Cd45.2*^±^, and *Cd45.2*^+*/*+^ mice, recombination activating enzyme 1 deficient (*Rag1*^*−/−*^) mice, and 2D2 transgenic mice (bearing the transgenic TCR specific for the recognition of the myelin oligodendrocyte glycoprotein (MOG)-derived peptide (MOG_35-55_ peptide) loaded on IA^b^ molecules; TCR_MOG_) were purchased from The Jackson Laboratory (Bar Harbor, ME). *Gpr43* knockout mice, (also called *Ffar2*^*−/−*^; C57BL/6N-Atm1Brd Ffar2tm1b(EUCOMM)Hmgu/JMmucd, RRID:MMRRC_047690-UCD) were obtained from the Mutant Mouse Resource and Research Center (MMRRC) at University of California at Davis, an NIH-funded strain repository (U42OD012210), and was donated to the MMRRC from Stephen Murray, The Jackson Laboratory. This mouse model was derived as part of the Knockout Mouse Production and Phenotyping Project (KOMP2) NIH NHGRI U54HG006332. All mouse strains were in the C57Bl/6 genetic background.

### EAE induction and evaluation

#### Immunisation and determination of the clinical score

Experimental groups were made by selecting mice randomly but maintaining the same proportions of males and females and paired by age. Mice displaying dwarfism or malformations were excluded. Experimental mice were s.c. immunised with 50 μg of MOG_35-55_ peptide (pMOG; Genetel Laboratories, Madison, WI) emulsified in complete Freund’s adjuvant (CFA; Invitrogen) supplemented with heat-inactivated *Mycobacterium tuberculosis* H37 RA (Difco Laboratories, Detroit, MI). In addition, mice received the i.p. administration of 500 ng pertussis toxin (Calbiochem, La Jolla, CA) on days 0 and 2. The clinical manifestation was assessed daily according to the following scoring criteria: 0, no detectable signs; 1, flaccid tail; 2, hind limb weakness or abnormal gait; 3, complete hind limb paralysis; 4, paralysis of fore and hind limbs; and 5, moribund or death. When indicated, supplementation of SCFAs (Fujifilm Wako chemicals, USA) was done in the drinking water at a final concentration of 250 mM and changed every three days; control mice received pH and sodium-matched water.

The animals were included in the study when they underwent successful pMOG-CFA immunisation, defined by the formation of a sub-cutaneous emulsion at the injection site. The animals were excluded when: (i) leakage of the emulsion was observed during injection, (ii) infections or diseases unrelated to the experiment were detected, or (iii) the animal died prematurely (usually 1 out of 200 experimental animals), avoiding collecting disease severity data. Of note, no additives (such as high calories “boost” and hydrogel) were administered after the onset of the disease, as the animals were able to feed and hydrate themselves throughout the whole course of the disease under our experimental conditions.

#### Isolation of CNS mononuclear cells

Mice were perfused through the left cardiac ventricle with cold PBS. The brain and spinal cord were dissected, and CNS tissue was minced into small pieces and digested by collagenase D (2.5 mg/mL; Roche Diagnostics) and DNase I (1 mg/mL; Sigma) at 37 °C for 45 min. Digested tissue was filtered through a 70 µm cell strainer obtaining a single cell suspension that was subjected to centrifugation in a density gradient made with Percoll (70%/30%). Mononuclear cells were removed from the interphase and resuspended in culture medium for further analysis. No blind protocol was carried out.

#### Isolation of mucosal lymphocytes

The isolation of intraepithelial (IEL) and lamina *propria* lymphocytes (LPL) from the colon was performed as previously described [28] with minor modifications. Briefly, colons were cut open longitudinally and washed with PBS to remove faeces and debris. Then, colons were incubated in HBSS containing 10 mM HEPES, 5 mM EDTA, 1 mM DTT and 5% FBS for 20 min at 37 °C, vortexed gently during the final 20 s, and the supernatant was collected. This procedure was repeated twice. The epithelial cells that were mechanically dissociated and collected in the supernatant were passed through 100 μm cell strainer, placed on a 50 mL Falcon tubes and collected as the fraction containing the IELs. The remaining colonic tissues were placed on gentleMACS™ tubes and digested in HBSS containing 10 mM HEPES, 0.5 mg/mL Collagenase D, 0.5 mg/mL DNase I grade II, 3 mg/mL liberase (Roche), and 5% FBS for 30 min at 37 °C on a shaking platform. After running gentleMACS™ dissociator program, the digested tissues containing the LPL were passed through 100 μm cell strainer. Leukocytes from both fractions were further enriched by Percoll gradient centrifugation (44%/67%).

### Antibodies and flow cytometry analysis

All analyses assessed live/dead discrimination using Zombie Aqua (ZAq) Fixable Viability kit (Biolegend). Spleens were minced until they reached a cell suspension and then red blood cells were lysed using ACK buffer (Ammonium Chloride 0.15 M; Potassium bicarbonate 0.01 M; Disodium EDTA 0.1 mM; pH 7.2–7.4). Fluorochrome-conjugated monoclonal antibodies (mAb) specific to mouse CD45 (clone 30-F11), CD19 (clone 6D5), PD-1 (clone 29.F.1A12), IL-10 (clone JES5-16E3), CXCR3 (clone CXCR3-A3), CD4 (clone GK-1.5), IFNγ (clone XMG1.2), IL-17 (clone TC11-18H10.1), TCRβ (clone B183983), TCRγδ (clone GL7), CD8α (clone 58-6,7), CD8β (clone YTS156.7.7), CD45.1 (clone A20) and CD45.2 (clone 104) were purchased from Biolegend and to mouse FoxP3 (clone FJK-16 s) from eBioscience.

For the immunostaining of GPR43, the rabbit anti-GPR43 antibody (AFR-032, Alomone labs) was directly used or pre-incubated with the antigenic peptide GPR43_301-314_ (RGAEETVEGTKTDR) used to develop the antibodies (in a mixture of 0.8 mg/mL antibody and 0.4 mg/mL peptide) for 30 min as a control to abolish the specific immunostaining. Secondary goat anti-rabbit IgG-PE (cat# 50-8036) was obtained from TONBO Biosciences.

For intracellular immunostaining, cells were first labelled with antibodies specific for cell-surface markers and then fixed and permeabilized with FoxP3 Fixation/Permeabilization kit (eBioscience). Afterwards, GPR43, Foxp3 and/or cytokine immunostaining was performed in permeabilized cells, followed by flow cytometry analysis. For analysis of cytokine production, cells were re-stimulated with 1 μg/mL ionomycin (Sigma) and 50 ng/mL PMA (Sigma) in the presence of 5 μg/mL brefeldin A (Invitrogen) for 3 h before immunostaining. All immunostainings were performed for 30 min at 4 °C. To quantify the absolute number of cells, 50 µL of 123 count eBeads (Thermofisher Scientific) was added to each sample prior to analysis by flow cytometry, and cell concentration was calculated using the following formula:

$$Cell\,Concentration (Cells/mL)=\frac{Cell\,Count\times eBead\,Volume}{eBead\,Count\times Cell\,Volume}\times eBead\,Concentration.$$

Data were collected with a FACSCanto II (BD) and results were analysed with FACSDiva (BD) and FlowJo software (Tree Star).

### In vitro culture

Colonic IEL and LPL isolated from WT mice were treated with 1.5 μM 4-CMTB (GPR43 agonist, Tocris) and then used for further analysis. For adoptive transfer experiments, 5 × 10^5^ 4-CMTB treated or untreated IEL were i.v. transferred into congenic recipient mice. For proliferation studies, IEL were labelled with Cell Trace Violet (CTV; Invitrogen) before the transfer and analysed at different time points as indicated.

### Real-time quantitative PCR

Total RNA was prepared from tissues or isolated cells and placed in cold PBS using TRIzol following the manufacturer’s instructions (Life Technologies). The RNA preparation was treated with DNase using the TURBO DNA-free kit (Ambion) and then used to synthesize cDNA catalysed by the M-MLV reverse transcriptase (Life Technologies). Quantitative gene expression analysis was performed using Brilliant II SYBR Green QPCR Master Mix (Agilent). Expression of target genes was normalised to the levels of *gapdh* transcripts and multiplied by an arbitrary factor. The following couple of primers were used: *gpr43* Forward 5’-ACA GTG GAG GGG ACC AAG AT-3’; *gpr43* Reverse 5’-GGG GAC TCT CTA CTC GGT CA-3’; *gpr41* Forward 5’-TTC TTG CAG CCA CAC TGC TC-3’; *gpr41* Reverse 5’-GCC CAC CAC ATG GGA CAT AT-3’; *gpr109a* Forward 5’-TCC AAG TCT CCA AAG GTG GT-3’; *gpr109a* Reverse 5’-TGT TTC TCT CCA GCA CTG AGT T-3’; *il17* Forward 5’-TTC ATC TGT GTC TCT GAT GCT-3’; *il17* Reverse 5´-AAC GGT TGA GGT AGT CTG AG-3’; *il22* Forward 5′-GAC AGG TTC CAG CCC TAC AT-3´; *il22* Reverse 5’-ATC GCC TTG ATC TCT CCA CT-3’; *ifng* Forward 5’- CGG CAC AGT CAT TGA AAG CCT A-3’; *ifng* Reverse 5´-GTT GCT GAT GGC CTG ATT GTC-3’; *il10* Forward 5’-GAA GAC AAT AAC TGC ACC CA-3’; *il10* Reverse 5’-CAA CCC AAG TAA CCC TTA AAG TC-3’; *il6* Forward 5’-AGG ATA CCA CTC CCA ACA GAC CT-3’; *il6* Reverse 5’-CAA GTG CAT CGT TGT TCA TAC-3’; *csf2* Forward 5’-ACC ACC GCG GAT TTC AT-3’; *csf2* Reverse 5-TCA TTA CGC AGG CAC AAA AG-3’; *gapdh* Forward 5’-TCC GTG TTC CTA CCC CCA ATG-3’; *gapdh* Reverse 5’-GAG TGG GAG TTG CTG TTG AAG-3’.

### SCFAs determination

21 serum samples and 19 faeces samples were collected for the quantitative analysis of SCFAs (acetic acid (C2), propionic acid (C3), butyric acid (C4), isobutyric acid (iC4), valeric acid (C5), isovaleric acid (iC5) and hexanoic acid (C6)) using a GC–MS method. A volume of 100–200 μL of serum samples was diluted in water containing labelled internal standards for each chain length (C2–C6). Faeces samples were homogenized in 5 mL water and then centrifuged. An aliquot of the supernatant was diluted in water containing labelled internal standards for each chain length (C2–C6). The free SCFAs were derivatized using methyl chloroformate in 1-propanol yielding propyl esters before subsequent liquid–liquid extraction into hexane and analysis on a SLB-5 ms (30 m × 0.25 mm × 1.0 μm) column followed by detection using GC-EI-MS in SIM-mode. The analytes were quantified using 8-point calibration curves.

### Transwells assays

Chemotaxis assays and analysis of lymphocyte subset migration were performed using 24-well Corning Transwells (5 μm pore size) as previously described [29, 30] with the following modifications. Briefly, freshly isolated IEL were allowed to recover from the isolation procedure by incubation in a chemotaxis medium (RPMI 1640 with 0.5% BSA from Sigma-Aldrich) for 1 h and then, the chemotaxis in response to increasing concentrations of propionate (30 μM to 3 mM) was allowed to proceed for 90 min. When indicated, after recovery time, IEL were pre-treated with 100 μM Propionate for 1 h, washed, and then, the chemotaxis in response to 300 ng/mL CXCL11 was allowed to proceed for 90 min.

### Analysis of faecal microbiome.

#### Collection of faecal samples and DNA extraction for metagenomic analysis

EAE was induced in C57BL/6 mice as indicated above (Sect. "Immunisation and determination of the clinical score"), and cecal samples were collected prior to the disease onset (5 dpi), at the peak of disease severity (15 dpi) and during the recovery phase (25 dpi). As a control, cecal samples were obtained before disease induction (0 dpi). Bacterial genomic DNA was extracted using Qiagen QIAamp DNA stool for whole-community DNA extraction. The 16S rRNA gene sequence and analysis were conducted in collaboration with the Alkek Center for Metagenomics and Microbiome Research (CMMR) at Baylor College of Medicine. Briefly, the 16S rDNA V4 region was amplified by PCR and sequenced in the MiSeq platform (Illumina) using the 2 × 250 bp paired-end protocol yielding pair-end reads overlapping almost completely. The primers used for amplification contain adapters for MiSeq sequencing and single-index barcodes so that the PCR products may be pooled and sequenced directly, targeting at least 10,000 reads per sample. The metagenome raw reads obtained from the sequencing of the faecal microbiome were deposited into Sequence Read Archive (SRA) bioproject PRJNA892997. An in-house pipeline was next used for read processing and analysis.

#### Inference of amplicon sequence variant from 16S amplicon sequencing

We employed the dada2 v1.24.0 [31] R package to process and infer amplicon sequence variants (ASV) from 16S amplicon sequences of stool samples. Raw reads were first quality trimmed and filtered using *filterAndTrim* function to remove low-quality reads and any reads that match the PhiX bacteriophage genome. We Kept all reads with an overall quality score above 30. Chimera detection was performed using the *removeBimeraDenovo* function with default parameters. The taxonomy assignment was carried out using the SILVA v138 reference database (updated to March 2021) [32].

#### Alpha diversity

We performed rarefaction curves using the vegan v2.6–2 R package to estimate the sample richness. Chao1 richness and Shannon diversity were calculated using phyloseq v1.40.0 [33].

#### Beta diversity

To infer the beta diversity of our samples, we carried out principal coordinates analysis (PCoA) based on unweighted UniFrac and the Bray–Curtis distance using phyloseq v1.40.0 [33]. The PERMANOVA (permutational multivariate analysis of variance) and betadisper tests were calculated with the vegan v2.6–2 R package. We also estimated the divergence of intestinal microbiota throughout the disease development.

#### Identifying differential abundance of taxa

To identify the differential abundance taxa with significant differences between DPI groups, we used the DESeq2 v1.36.0 R package [34]. Differentially abundant taxa were determined using a *p*-value < 10–3 and adjusted *p*-value < 0.01 thresholds.

### Statistical analyses and sample size estimation

The sample size was estimated with the mean and dispersion obtained from preliminary data using the sample size calculator: https://www.stat.ubc.ca/~rollin/stats/ssize/n2.html. A power of 80% was assumed. All values are expressed as the mean ± SEM. Statistical analysis was performed with two-tailed unpaired Student’s *t*-test when comparing only two groups and with one-way ANOVA followed by Dunnett’s or Tukey's *post-hoc* test when comparing more than two groups with only one variable (treatment or genotype). To analyse differences in experiments comparing different genotypes and different treatments, two-way ANOVA followed by Sidak's *post-hoc* test was performed. All analyses were carried out using the GraphPad Prism 6 Software. *p*-values < 0.05 were considered significant.

## Results

### Central nervous system autoimmunity involves a reduction in the levels of short-chain fatty acids and changes in the inflammatory profile of the colonic mucosa

**Fig. 1:**
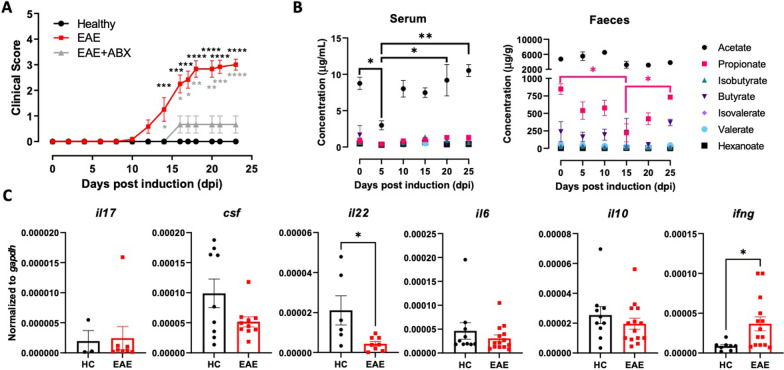
Reduced levels of SCFAs are associated with a pro-inflammatory environment in the colonic mucosa during EAE. **A** EAE was induced in untreated WT (EAE, red) or Antibiotic-treated (grey, EAE + ABX: Antibiotic treatment from dpi 5 to 25; ampicillin 1 g/L and streptomycin 1 g/L) mice by immunisation with pMOG in CFA followed by pertussis toxin injection and then, the disease severity was determined throughout the time-course of the disease development. Values represent mean ± SEM from 7–8 mice per group. **B** Systemic and intestinal levels of SCFAs were determined by gas chromatography coupled to mass spectrometry in serum and faeces, respectively, at various time points during the disease development. Values represent mean ± SEM from 4–5 mice per group. **C** Distal colons sections were isolated at 15 dpi, and the levels of cytokine transcripts were analysed by qRT-PCR. The levels of *gapdh* transcripts were used as a housekeeping. Data were obtained from 5 to 14 mice per group. Each symbol represents data obtained from an individual mouse. Mean ± SD are indicated. *, p < 0.05; **, p < 0.01; ***, p < 0.001; ****, p < 0.0001 by (A and B) one-way ANOVA followed by Tukey’s post-hoc test, or (C) student’s *t*-test. (B) Black asterisks correspond to comparisons between EAE and healthy groups, whilst grey asterisk correspond to comparisons between EAE and EAE + ABX groups

Previous studies have shown that the lack of commensal microbiota attenuates the development of CNS autoimmunity [15, 35, 36]. Nevertheless, it has also been described that the composition of the commensal microbiota varies among different animal facilities [37, 38]. To study whether the commensal microbiota affects the development of CNS autoimmunity in our experimental conditions, we analysed how the depletion of the microbiota induced by antibiotics (ABX) impacts the severity of EAE development. Concordant with previous results [36], EAE severity was strongly reduced in animals treated with ABX (Fig. 1A), indicating that the commensal microbiota somehow contributes to EAE manifestation. To characterise the microbiome profiles during EAE development, metagenomic sequencing of the variable V4 region of the prokaryotic 16S ribosomal RNA gene present in faeces was performed. Our analysis revealed an increase in alpha diversity at the peak of EAE severity (15 dpi) when calculated by Chao1 (Additional file 1: Fig. S1A, left panel). Although no differences were observed in the Shannon-calculated alpha diversity (Additional file 1: Fig. S1A, right panel) or beta diversity (Additional file 1: Fig. S1C) throughout the disease development. Furthermore, the heterogeneity in the community composition was significantly decreased at 15 dpi compared with healthy mice (Additional file 1: Fig. S1B). Moreover, the analysis of faecal microbial composition at the Phylum level shows a reduction of the percentage of ASV corresponding to the Firmicutes phylum and an increase in the Bacteroidetes phylum at the peak of the disease manifestation (Additional file 1: Fig. S1D-E). In addition, the analysis of the top three microbial composition at the Family level indicates that the relative abundance of the Firmicutes *Erysipelotrichaceae* is reduced (Additional file 1: Fig. S1F), whilst the Bacteroidetes *Prevotellaceae* is increased at 15 dpi compared to healthy mice (Additional file 1: Fig. S1F). Interestingly, both phyla have been involved in the production of SCFAs [39, 40]. According to previous findings indicating a regulatory role of SCFAs on CNS autoimmunity [25–27], we next addressed the possibility that these metabolites mediate the effect of microbiota in EAE manifestation. Since oral SCFAs administration decreases EAE severity [25–27, 41, 42], we aimed to test whether EAE development involves changes in the endogenous production of SCFAs by the commensal microbiota. Our results show a selective reduction in the levels of propionate in the faeces during the peak of EAE manifestation and decreased levels of acetate in the serum prior to the disease onset (Fig. 1B), thus suggesting a role of intestinal and systemic SCFAs in the regulation of CNS autoimmunity. According to the fundamental role of T-cells in CNS autoimmunity [7] and the role of SCFAs in the regulation of inflammation [18–23], we next evaluated whether the local reduction of propionate levels is related to changes in the profile of T-cell-derived cytokines in the colonic mucosa at the peak of EAE manifestation. The results show that the decrease in propionate levels was accompanied by increased transcription of *ifng* and reduced transcription of *il22* in the colonic mucosa (Fig. 1C). Since intestinal IL-22 is involved in promoting barrier function [20], and IFNγ constitutes the main effector cytokine of Th1 and CD8^+^ T-cells, these results suggest that SCFAs changes observed in the peak of EAE manifestation may be related to reduced gut barrier function and increased permeability of the colonic epithelial layer accompanied by the local activation of Th1 and/or CD8^+^ T-cells responses.

### Propionate induces an anti-inflammatory profile in mucosal TCRαβ^+^ T-cells through the stimulation of GPR43

**Fig. 2:**
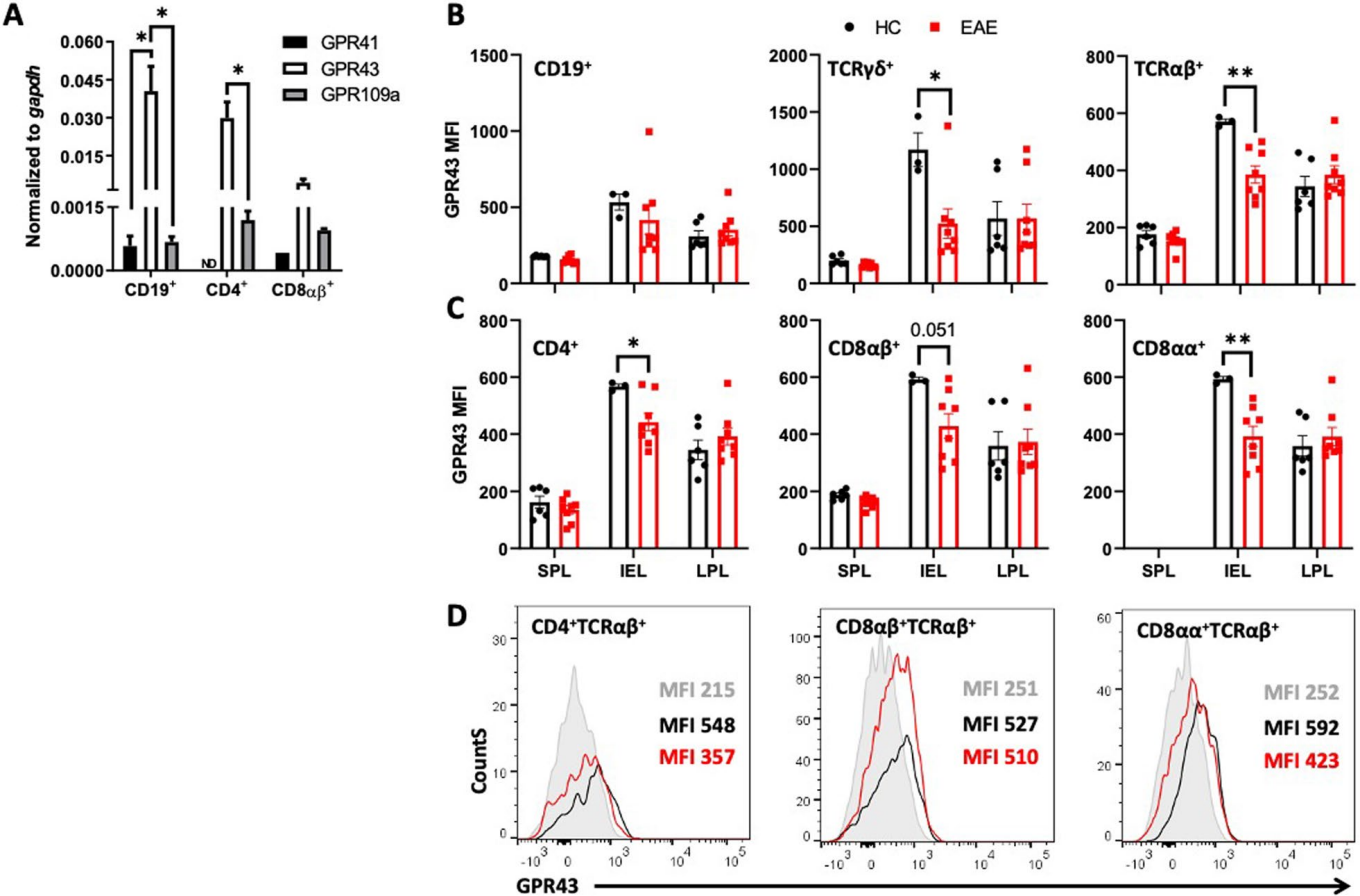
GPR43 expression is reduced in IEL upon EAE. **A** Levels of transcripts encoding GPR41, GPR43 and GPR109a were determined by RTqPCR and normalised with the levels of *gapdh* on CD19^+^ B-cells, CD4^+^ TCRαβ^+^ and CD8αβ^+^ TCRαβ^+^ T-cells isolated from spleen of healthy mice. Values represent mean ± SEM from 9 mice per group. ND: not detected. (B-D) GPR43 expression at protein level was determined on **B** CD19^+^ B cells, TCRγδ^+^ and TCRαβ^+^ T-cells and **C** on CD4^+^, CD8αβ^+^ and CD8αα^+^ subsets of TCRαβ^+^ T-cells from spleen (SPL), intraepithelial lymphocytes (IEL) and lamina *propria* lymphocytes (LPL) obtained from healthy (black) or EAE mice at 15dpi (red). Values are the mean fluorescence intensity (MFI) associated to GPR43 immunostaining. Data were obtained from 6–8 mice per group. Each symbol represents data obtained from an individual mouse. Mean ± SD are indicated. **D** Representative histogram of GPR43 expression analysed by flow cytometry. Control (filled grey), healthy mice (black) and EAE mice (red). *, p < 0.05; **, p < 0.01; by one-way ANOVA followed by Tukey’s post-hoc test (A) or unpaired Student’s t-test (B and C)

To evaluate whether changes in the levels of SCFAs and the pro-inflammatory profile of cytokines observed in the colonic mucosa during the peak of EAE manifestation were connected, we next determined the expression of SCFAs receptors in lymphocytes. Accordingly, we first analysed the levels of transcripts encoding GPR41, GPR43, and GPR109a in CD19^+^ B cells and CD4^+^ and CD8^+^ T-cells in homeostatic conditions. We observed that the transcript encoding GPR43 was much higher expressed than transcripts encoding GPR41 and GPR109a in all the lymphocyte subsets analysed (Fig. 2A). For this reason, we next focused further analyses on the expression of surface GPR43 on lymphocytes upon EAE development. To this end, the surface GPR43 expression, at the protein level, was determined on intraepithelial lymphocytes (IEL) or lamina *propria* lymphocytes (LPL) isolated from the colonic mucosa of EAE mice at the peak of disease manifestation or from healthy controls. GPR43 was also analysed on splenic lymphocytes to have an insight of the systemic expression. The results show that GPR43 expression was selectively down-regulated during EAE development on the IEL (Fig. 2B). Conversely, GPR43 expression was not affected on LPL or splenic lymphocytes upon EAE (Fig. 2B). Interestingly, GPR43 down-regulation was observed on the IEL TCRαβ^+^ and TCRγδ^+^ T-cell (Fig. 2B). Among TCRαβ^+^ IEL, GPR43 was down-regulated on the CD4^+^, CD8αβ^+^, and CD8αα^+^ T-cells upon EAE (Fig. 2C, D).

To determine whether the down-regulation of GPR43 expression on TCRαβ^+^ IEL resulted from the reduction in the levels of intestinal SCFAs, we treated healthy mice with oral administration of acetate, propionate, or butyrate for two weeks, and the GPR43 expression was analysed on TCRαβ^+^ IEL. The results show that the oral treatment with either propionate or butyrate induced the up-regulation of GPR43 expression on TCRαβ^+^ IEL, specifically in the CD8αβ^+^ and CD8αα^+^ subpopulations (Additional file 1: Fig. S2A, B). Thus, these results indicate that intestinal propionate and butyrate regulate the expression of IEL TCRαβ^+^ T-cells. According to this conclusion, we observed that GPR43 expression was up-regulated on IEL TCRαβ^+^ T-cells at the recovery phase of EAE development (Additional file 1: Fig. S2C–E), a time-point (25 dpi) in which the levels of faecal propionate are recovered (Fig. 1B).

**Fig. 3:**
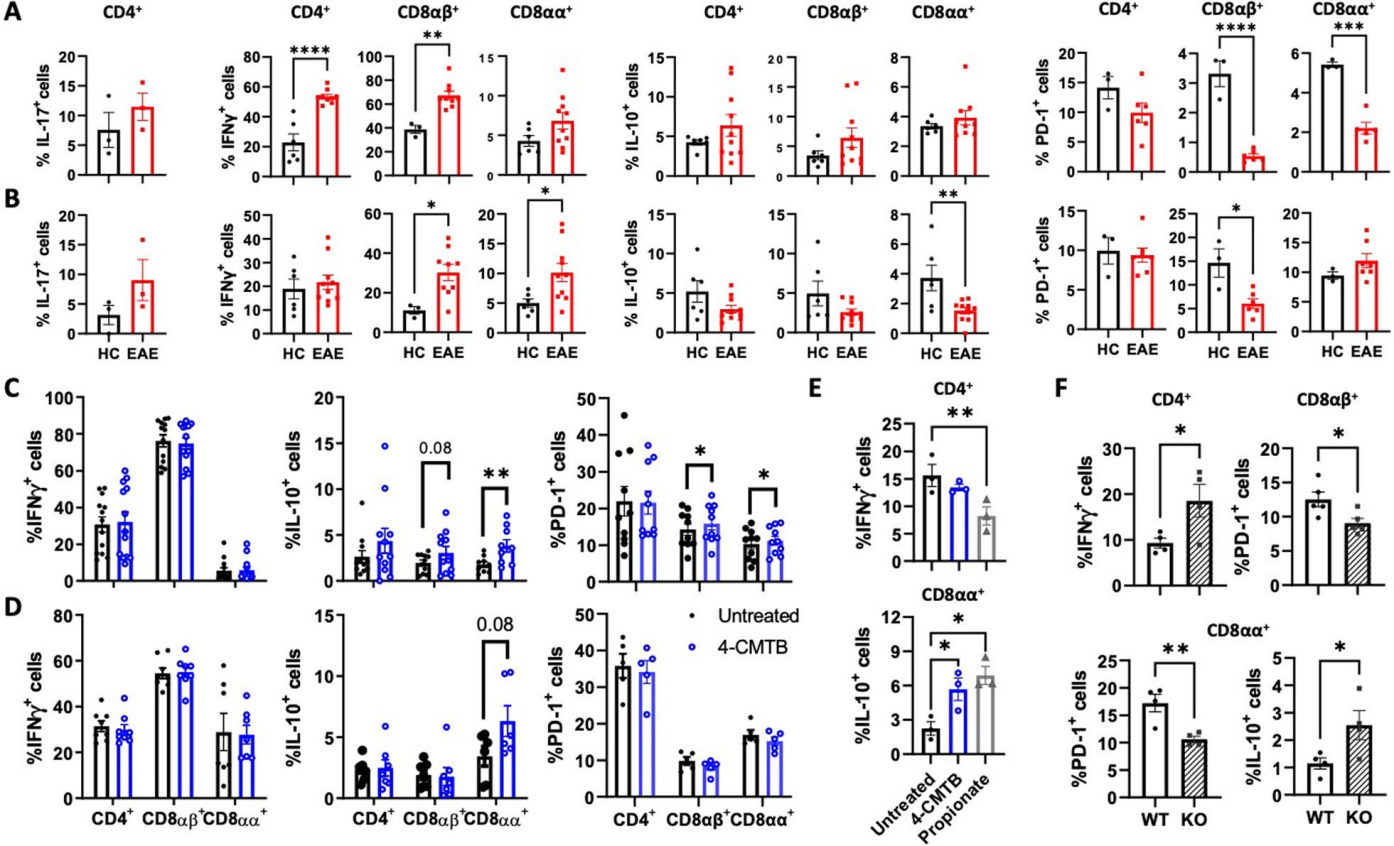
Colonic TCRαβ^+^ T-cells acquire an inflammatory profile upon EAE, which might be reverted by GPR43 stimulation. **A** IEL and **B** LPL were isolated from healthy (black) or EAE mice (red) at 15 dpi and then re-stimulated with PMA/Iono/BFA for 3 h to analyse the expression of pro- and anti-inflammatory surface molecules and cytokines in CD4^+^, CD8αα^+^ and CD8αβ^+^ TCRαβ^+^ T-cells. Values are the percentage of T-cells producing IL-17, IFNγ, IL-10 or expressing PD-1. Each symbol represents data obtained from an individual mouse. **C** and **E** IEL and **D** LPL were isolated from the colon of WT healthy mice and then ex vivo stimulated with PMA/Iono/BFA in the presence of 1.5 μM 4-CMTB (blue symbols), 100 μM Propionate (grey symbols) or left untreated (black symbols). **F** IEL were isolated from the colon of WT (open bars) or GPR43 knockout (KO; grey-filled bars) healthy mice and then ex vivo stimulated with PMA/Iono/BFA. Then intracellular cytokine staining was analysed by flow cytometry. Values are the percentage of T-cells producing IL-17, IFNγ, IL-10 or expressing PD-1. Each symbol represents data obtained from an individual mouse. **A**–**F** Mean ± SD are indicated. Data were obtained from **A**–**B** 3–8, **C**–**D** 14, or **E**–**F** 3–5 mice per group. *, p < 0.05; **, p < 0.01; ***, p < 0.001; ****, p < 0.0001 by paired (**C** and **D**) or unpaired (**A**, **B** and **F**) Student’s t-test, or by one-way ANOVA followed by Dunnett’s post-hoc test (**E**)

To evaluate whether the reduction of propionate levels and GPR43 down-regulation were associated with changes in the inflammatory profile of mucosal lymphocytes at the peak of EAE manifestation, IFN-γ and IL-17 were analysed in colonic IEL and LPL isolated from EAE and healthy mice by intracellular cytokine staining. The results show a significant increase in IFN-γ production in the CD4^+^ and CD8αβ^+^ subpopulations of TCRαβ^+^ IEL and in the CD8αβ^+^ and CD8αα^+^ subpopulations of TCRαβ^+^ LPL upon EAE (Fig. 3A, B). No changes were observed in IL-17 production by colonic CD4^+^ TCRαβ^+^ either from the IEL or the LPL compartments (Fig. 3A, B). To expand our analysis to anti-inflammatory molecules, we also analysed IL-10 production and the surface expression of PD-1 in the same lymphocyte subsets during EAE. We observed a reduction in the production of IL-10 by CD8αα^+^ TCRαβ^+^ LPL and decreased PD-1 expression on CD8αβ^+^ and CD8αα^+^ from TCRαβ^+^ IEL and on CD8αβ^+^ TCRαβ^+^ LPL (Fig. 3A, B). These results together show that concomitant to the reduction of SCFAs-signalling on mucosal T-cells, various colonic TCRαβ^+^ subsets experienced phenotypic changes involving higher pro-inflammatory cytokine production (IFN-γ) and lower levels of anti-inflammatory molecules (IL-10 and PD-1) at the peak of EAE manifestation.

To address whether reduced SCFAs-signalling was responsible for the phenotypic changes observed in colonic IEL and LPL TCRαβ^+^ T-cells during EAE, we next isolated these lymphocytes and stimulated them ex vivo with either propionate or a selective GPR43-agonist (4-CMTB) and the pro-inflammatory/anti-inflammatory profile was determined. Although we did not observe changes in the extent of IFN-γ production by TCRαβ^+^ IEL when stimulated with 4-CMTB (Fig. 3C), we noticed a significant reduction in the frequency of CD4^+^ TCRαβ^+^ IEL producing IFN-γ when stimulated with propionate (Fig. 3E). Furthermore, we observed increased expression of PD-1 and enhanced production of IL-10 on CD8^+^ TCRαβ^+^ IEL when stimulated with 4-CMTB or propionate (Fig. 3C, E). No significant changes were detected in PD-1, IL-10, or IFN-γ expression on TCRαβ^+^ LPL when stimulated with the selective GPR43-agonist (Fig. 3D). To evaluate whether the down-regulation of GPR43 observed on IEL TCRαβ^+^ T-cells (Fig. 2C, D) was enough to promote a pro-inflammatory profile in these cells, we evaluated the inflammatory/anti-inflammatory profile of IEL TCRαβ^+^ T-cells isolated from *gpr43* knockout mice. The results show that the GPR43 deficiency favours a higher production of IFN-γ in CD4^+^ TCRαβ^+^ T-cells, lower surface expression of PD-1 on CD8αβ^+^ and CD8αα^+^ TCRαβ^+^, although higher IL-10 production on CD8αα^+^ TCRαβ^+^ (Fig. 3F). These results suggest that the GPR43 down-regulation per se might favour a pro-inflammatory behaviour in IEL TCRαβ^+^ T-cells.

To gain more robust evidence about the role of SCFAs in acquiring anti-inflammatory features in colonic TCRαβ^+^ lymphocytes, we next analysed the pro-inflammatory/anti-inflammatory profile of mucosal T-cells obtained from EAE mice treated with oral administration of acetate, propionate, or butyrate. As described before [25–27], all the SCFAs tested ameliorated the disease manifestation (Additional file 1: Fig. S3A). Of note, only acetate, but not propionate or butyrate, affected the water consumption rate (Additional file 1: Fig. S3B). In contrast to acetate and butyrate, the results show a selective effect of propionate increasing the production of IL-10 as well as an enhanced number of CD4^+^, CD8αβ^+^, and the total number of TCRαβ^+^ lymphocytes in the IEL compartment, which was accompanied by a significant attenuation on the disease severity (Additional file 1: Fig. S3A, C). However, in contrast to the results obtained using the ex vivo approach (Fig. 3D), the oral administration of propionate induced a significant increase in IL-10 production and enhanced PD1 expression on some subsets of LPL Additional file 1: Fig. S3D), thus suggesting that propionate act indirectly on LPL favouring an anti-inflammatory phenotype on these cells. Altogether, these results indicate that the reduction of SCFAs levels observed at the peak of EAE manifestation promotes the acquisition of a pro-inflammatory behaviour on colonic TCRαβ^+^ lymphocytes.

### T-cells infiltrate the colon before reaching the CNS upon EAE development

**Fig. 4:**
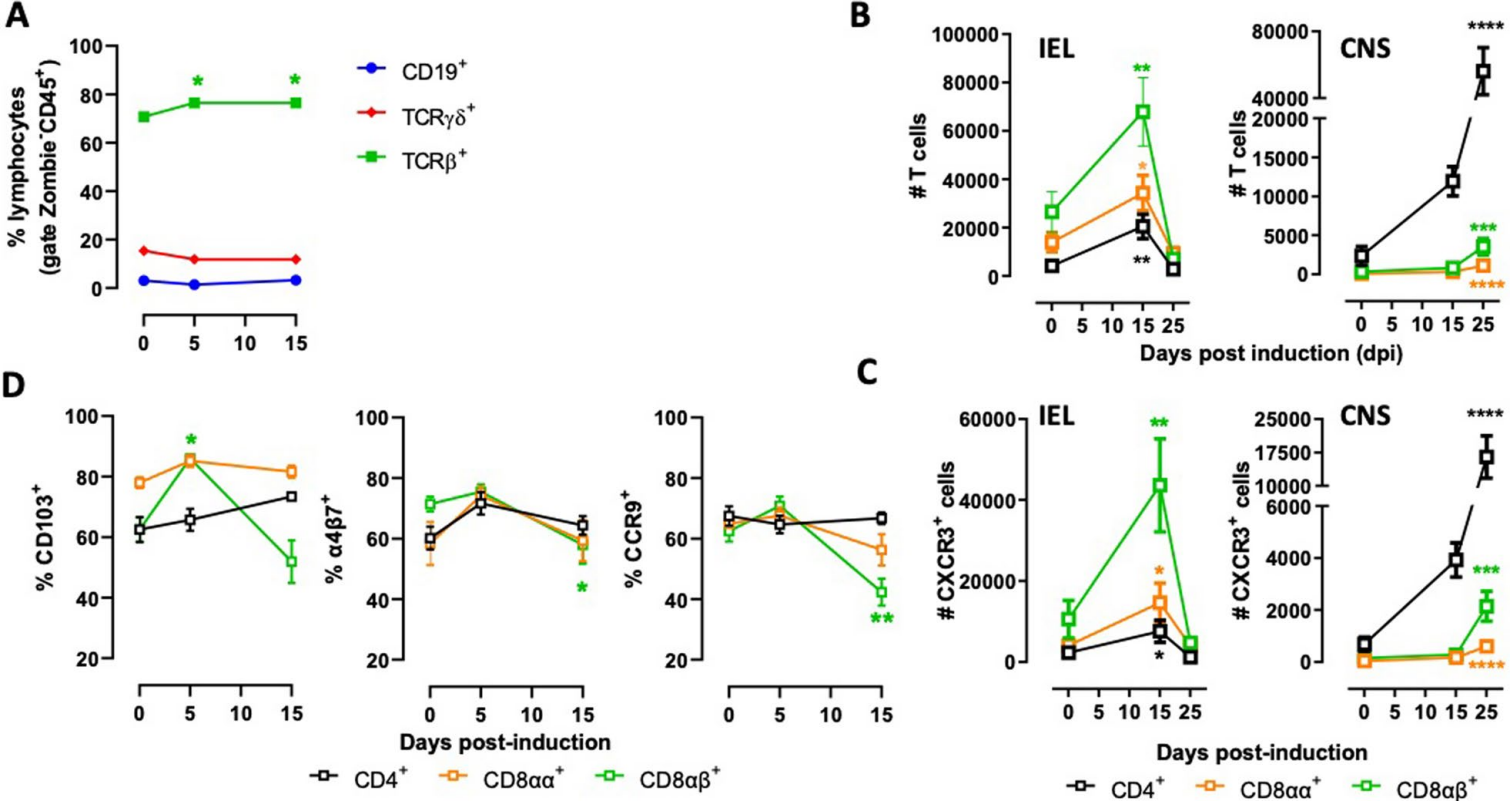
T-cells accumulate in the colonic intraepithelial compartment prior CNS infiltration. **A** Colonic IEL were isolated from EAE mice at indicated time points and the frequency of CD19^+^ B-cells, TCRγδ^+^ and total TCRαβ^+^ T-cells was analysed. **B**, **C** Colonic IEL and MNCs from CNS were isolated from EAE mice at indicated time points and the absolute number of **B** T-cell subsets CD4^+^, CD8αα^+^ and CD8αβ^+^ TCRαβ and **C** CXCR3 expressing T-cells was determined. **D** Percentage of CD4^+^, CD8αα^+^ and CD8αβ^+^ TCRαβ T-cells expressing CD103, α4β7 and CCR9. **A**–**D** Data were obtained from 6 to 12 mice per group. Values represent the mean ± SD. *, p < 0.05; **, p < 0.01; ***, p < 0.001; ****, p < 0.0001 by One-way ANOVA followed by Dunnett’s post-hoc test comparing frequencies of each cell type at different time points with the control condition (0 dpi)

To gain a deeper insight into the role of IEL during CNS autoimmunity, we next analysed the dynamics of different lymphocyte populations along different time points during EAE development. To this end, we isolated colonic IEL from mice before EAE induction, during the disease induction stage (5 dpi), and at the peak of EAE manifestation (15 dpi) and analysed the composition of lymphocyte populations. The results show that IEL are composed mainly of TCRαβ^+^ T-cells in healthy mice (~ 70%), and the relative amount of these cells slightly increased during EAE development (Fig. 4A). Conversely, TCRγδ^+^ T-cells constitute less than 20%, and B cells were barely detectable in IEL from healthy mice or after EAE induction at all the time points analysed (Fig. 4A). To determine whether IEL TCRαβ^+^ T-cells move freely to other tissues or maintain their epithelial tropism during healthy conditions, we performed in vivo experiments to determine the tissue preferences of these cells upon homeostatic conditions. For this purpose, IEL were isolated from *Cd45.1*^+*/*+^ donor mice and i.v. transferred into congenic (*Cd45.2*^+*/*+^) lymphopenic mice (*Rag1*^*−/*−^), and the arrival of donor cells was tracked along the time (Additional file 1: Fig. S4A). The results show that donor IEL TCRαβ^+^ T-cells were preferentially retained in the colonic epithelium (Additional file 1: Fig. S4B). Of note, the IEL TCRαβ^+^ T-cells infiltrated in the different tissues presented a similar extent of homeostatic proliferation, ruling out the possibility that a higher percentage of donor IEL TCRαβ^+^ T-cells in the colonic epithelium was due to an enhanced extent of proliferation in this tissue (Additional file 1: Fig. S4C, D). Thus, these results indicate that under homeostatic conditions, IEL TCRαβ^+^ T-cells prefer to stay in the epithelial niche and not move to other tissues. We next addressed whether IEL TCRαβ^+^ T-cells move from the colonic epithelium upon CNS autoimmunity. To this end, we analysed the composition of TCRαβ^+^ T-cells in the IEL and CNS at key time points during EAE development. We observed that the number of cells of the three TCRαβ^+^ IEL subsets, CD4^+^, CD8αα^+^, and CD8αβ^+^ T-cells, presented a significant increase at 15 dpi (Fig. 4B, left panel). Conversely, the number of these three subsets of TCRαβ^+^ T-cells presented a sharp reduction at 25 dpi (Fig. 4B, left panel). Concomitantly, CD4^+^, CD8αα^+^, and CD8αβ^+^ T-cells infiltrated the CNS at 25 dpi (Fig. 4B, right panel). These results suggest that TCRαβ^+^ T-cells present in the colonic epithelium during the peak of EAE manifestation leave the gut mucosa to reach the CNS during the recovery stage of the disease (25 dpi). Since the chemokine receptor CXCR3 has been involved in lymphocyte infiltration into the CNS upon neuroinflammation [11, 43–47], we determined the dynamics of the expression of this receptor on TCRαβ^+^ T-cells obtained from the colonic epithelium and CNS during different stages of EAE development. Interestingly, we observed a significant increase of CXCR3^+^ TCRαβ^+^ T-cells in the colonic epithelium at 15 dpi, followed by a substantial decrease at 25 dpi (Fig. 4C, left panel). The reduction of CXCR3^+^ TCRαβ^+^ T-cells in the colonic mucosa was accompanied by a significant infiltration of CXCR3^+^ TCRαβ^+^ T-cells into the CNS during the recovery stage (Fig. 4C, right panel). Thus, these results suggest that TCRαβ^+^ T-cells accumulate in the colonic epithelium at the peak of EAE manifestation and then move to the CNS by a mechanism involving CXCR3-mediated migration. Next, to determine whether this dynamic of T-cell subsets is associated with changes in surface molecules involved in intestinal tropism, we analysed the expression of the integrins α4β7, CD103, and the chemokine receptor CCR9 [48–50]. The results show that all three surface molecules associated with gut tropism were down-regulated on CD8αβ^+^ T-cells during the peak of EAE manifestation (Fig. 4D). CCR9, α4β7, and CD103 expression were not significantly altered on CD8αα^+^ and CD4^+^ T-cells during the time points in which they were evaluated (Fig. 4D). Of note, the dynamics of lymphocyte composition and their surface expression of CXCR3, CCR9, CD103, and α4β7 were also analysed on LPL at key time points during EAE development; however, the results show no relationship with those dynamics observed in IEL (Additional file 1: Fig. S5). For these reasons, further experiments analysing the dynamics of mucosal TCRαβ^+^ T-cells were focused on IEL. Altogether, these results suggest that IEL TCRαβ^+^ T-cells undergo a change of tissue tropism during the peak of EAE manifestation and then leave the gut mucosa to reach the CNS during the recovery stage of the disease.

### GPR43 stimulation retains TCRαβ T-cells in the colonic epithelium

**Fig. 5:**
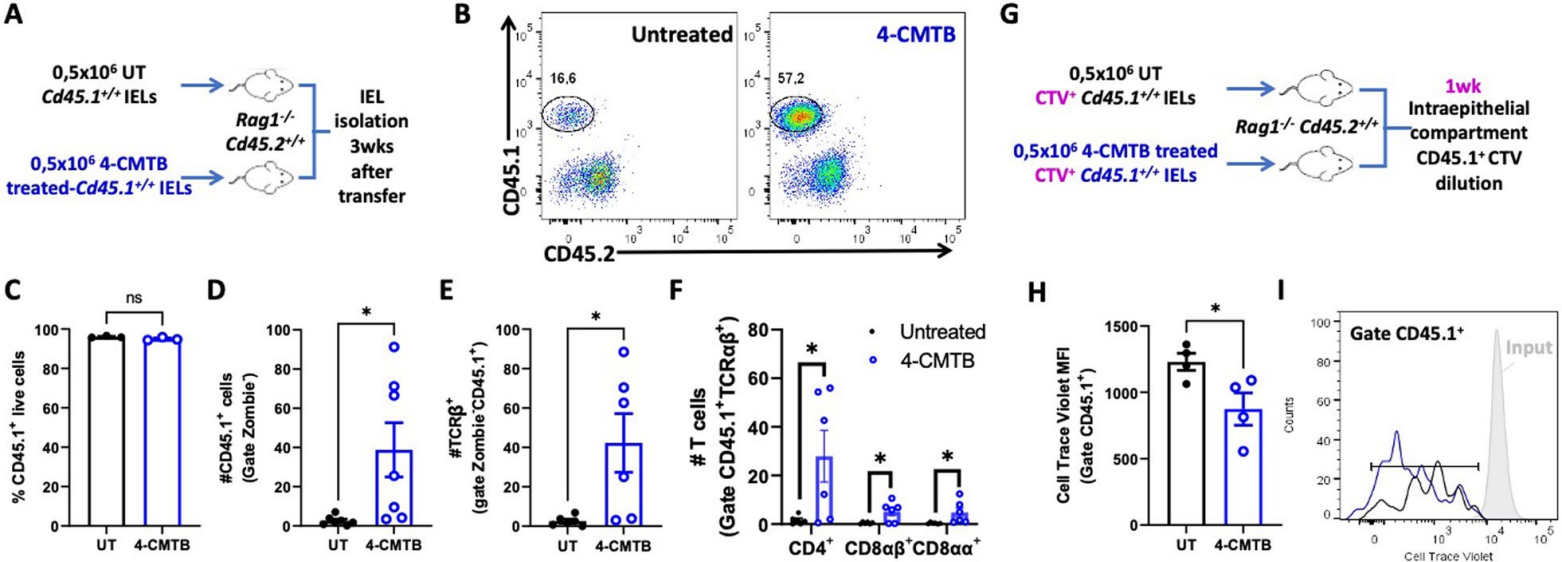
GPR43 stimulation enhances the retention of TCRαβ^+^ IEL into the colonic epithelium. **A**–**F** IEL were isolated from *Cd45.1*^+*/*+^ congenic mice and then treated ex vivo with 1.5 μM 4-CMTB (blue) or left untreated (black) for 1 h. Afterwards, cells were i.v. transferred into *Rag1*^*−/−*^* Cd45.2*^+*/*+^recipient mice, and 3 weeks later the number of cells expressing CD45.1, TCRβ, CD4, CD8αα, CD8αβ was analysed in IEL. **A** Schematic illustration of *Cd45.1*^+*/*+^ IEL adoptive transfer into *Rag1*^*−/−*^ recipients. **B** Representative dot plots analysing the percentage of donor and recipient cells. **C** Percentage of CD45.1^+^ live cells detected in *Rag1*^*−/−*^ recipients. Absolute numbers of CD45.1^+^ (**D**), TCRβ^+^ (**E**) and donor T-cells subsets (**F**) isolated from the intraepithelial compartment of *Rag1*^*−/−*^ recipient mice. Data were obtained from 6–7 mice per group. Each symbol represents data obtained from an individual mouse. (G-I) IEL were isolated from *Cd45.1*^+*/*+^ congenic mice, loaded with CTV, treated or not with 1.5 μM 4-CMTB for 1 h and then transferred into *Rag1*^*−/−*^* Cd45.2*^+*/*+^recipient mice. One week later the extent of T-cell proliferation was analysed in IEL. **G** Schematic illustration. **H**, **I** The proliferation of donor cells was determined as the dilution of CTV-associated fluorescence in CD45.1^+^ IEL. **H** Quantification of the MFI associated to CTV immunostaining. Each symbol represents data obtained from an individual mouse. **C**, **D**, **E**, **F**, **H** Mean ± SD are indicated. *, p < 0.05; by unpaired Student’s t-test. ns, non-significant. **I** Representative histogram of CTV associated fluorescence

Our results presented above show that GPR43 is downregulated on IEL TCRαβ^+^ T-cells, and the levels of intestinal propionate undergo a substantial reduction during the peak of EAE manifestation, followed by the exit of IEL TCRαβ^+^ T-cells from the colonic epithelium. To explore whether GPR43-signalling is involved in the exit of IEL TCRαβ^+^ T-cells from the gut mucosa, we next performed a set of in vivo experiments in which GPR43 was pharmacologically stimulated in IEL TCRαβ^+^ T-cells and the retention of these cells in the colonic epithelium was determined. We first stimulated IEL with a selective GPR43 agonist ex vivo, and then, they were transferred into congenic *Rag1*^*−/−*^ recipient mice, and the extent of T-cells infiltration into the colonic epithelium was evaluated under homeostatic conditions (Fig. 5A). The results show that GPR43-stimulation of IEL promotes a substantial increase in the number of all the TCRαβ^+^ T-cell subsets in the colonic epithelium, including CD4^+^, CD8αα^+^, and CD8αβ^+^ T-cells (Fig. 5 B-F). This increase was not due to a different survival of the transferred cells (Fig. 5C). In addition, we evaluated whether GPR43-stimulation affected the proliferation of IEL TCRαβ T-cells. To this end, we performed similar experiments but loading IEL with cell trace violet (CTV) ex vivo before the treatment with 4-CMTB (Fig. 5G). We observed that GPR43-stimulation resulted in a slight increase in the proliferation of donor IEL in the colonic epithelium (Fig. 5H, I). These results indicate that GPR43-signalling in TCRαβ^+^ T-cells favours their retention in the colonic epithelium and a slight increase in the proliferation rate under homeostatic conditions.

**Fig. 6:**
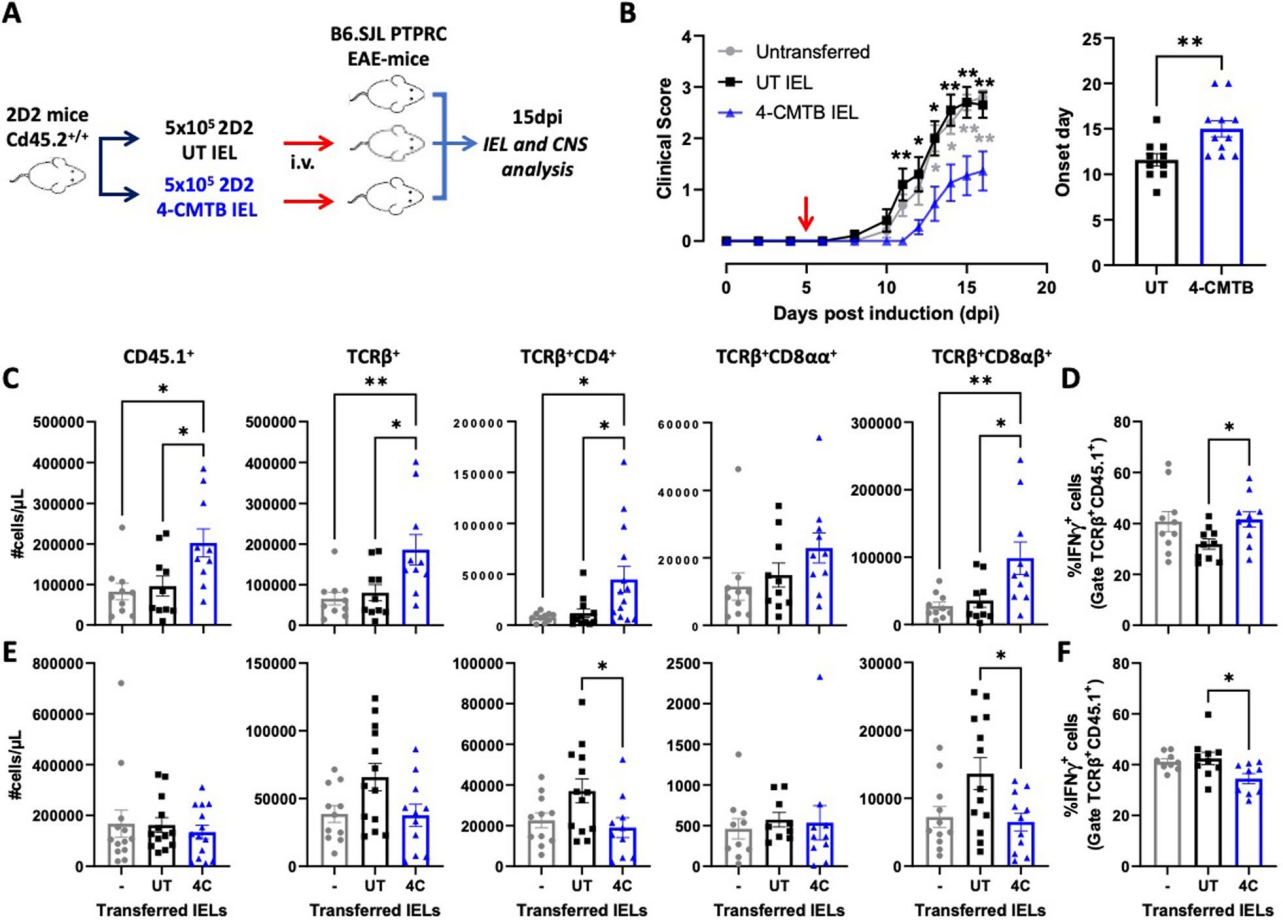
GPR43-stimulation of IEL exerts an anti-inflammatory effect in vivo, which attenuates EAE manifestation. IEL isolated from 2D2 transgenic *Cd45.2*^+*/*+^ congenic mice were left untreated (black) or treated with 1.5 μM 4-CMTB (blue) for 1 h and then i.v. transferred into *Cd45.1*^+*/*+^ congenic mice undergoing EAE at 5 dpi. **A** Schematic illustration of the experimental design. **B** Disease severity and the onset were determined. In the left panel, values represent mean ± SEM. In the right panel, each symbol represents data obtained from an individual mouse. Mean ± SD are indicated. **C** and** E** Absolute number of CD45.1^+^ total cells or CD45.1^+^ in specific T-cell subsets isolated from IEL (**C**) or CNS (**E**) at 15 dpi. **D** and** F** Percentage of CD45.1^+^ TCRαβ^+^ cells producing IFNγ isolated from IEL (**D**) or CNS (**F**) at 15 dpi. **C**–**F** Mean ± SD are indicated. **A**–**F** Data were obtained from 10–11 mice per group. Each symbol represents data obtained from an individual mouse. *, p < 0.05; **, p < 0.01 by (**B** left panel, **C**, **D**, **E**, **F**) one-way ANOVA followed by Tukey’s post-hoc test or by (**B** right panel) unpaired Student’s t-test. (**B** left panel) Black asterisks correspond to comparisons between UT-IEL and 4-CMTB IEL, whilst grey asterisks correspond to comparisons between non-transferred and 4-CMTB IEL groups

Afterwards, we conducted a second set of in vivo experiments to determine the effect of GPR43-stimulation in the retention of TCRαβ^+^ T-cells in the colonic epithelium, but now, in the context of CNS autoimmunity. Accordingly, we isolated IEL from transgenic 2D2 mice (that harbour TCRαβ^+^ T-cells expressing a transgenic TCR specific to pMOG [51]), which were stimulated or not with the selective GPR43 agonist ex vivo, and then i.v. transferred into congenic recipient mice undergoing EAE (Fig. 6A). Importantly, we observed that mice receiving the transfer of 4-CMTB-stimulated 2D2 IEL presented a substantial reduction in the disease severity and a delay in the onset of EAE, whilst the transfer of untreated 2D2 IEL did not exert any effect on disease manifestation (Fig. 6B). Furthermore, the transfer of GPR43-stimulated 2D2 IEL in EAE mice induced higher retention of endogenous TCRαβ^+^ T-cells in the colonic epithelium (Fig. 6C) and an enhanced frequency of IFN-γ-producing T-cells in this tissue (Fig. 6D). Conversely, mice receiving the transfer of 4-CMTB-stimulated 2D2 IEL displayed a lower extent of endogenous TCRαβ T-cells infiltration into the CNS (Fig. 6E) and reduced IFN-γ-producing T-cells frequency (Fig. 6F). These results indicate that GPR43-stimulation on IEL TCRαβ^+^ T-cells retains inflammatory T-cells in the gut mucosa, thus avoiding their subsequent infiltration into the CNS of EAE mice, dampening the disease severity.

**Fig. 7:**
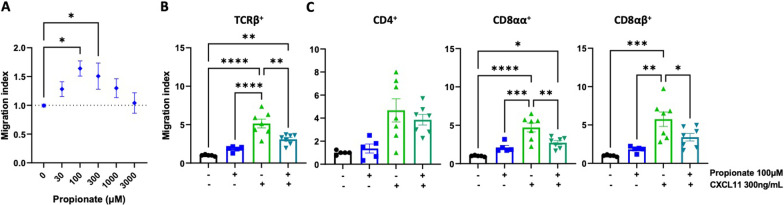
Propionate attenuates CXCL11-mediated migration of IEL TCRαβ^+^ T-cells. **A** The ability of IEL to migrate towards increasing concentrations of propionate was determined in transwell assay. Values represent mean ± SEM from 5 to 7 mice per group. **B**, **C** IEL were isolated from WT mice, stimulated with 100 μM propionate for 1 h and then the migration of TCRαβ^+^ T-cell subsets in response to 300 ng/mL CXCL11 was determined in transwell assay. Values are the number of TCRαβ^+^ T-cells on the bottom chamber normalised to the control condition (non-treated). Data were obtained from 7 to 10 mice per group. Each symbol represents data obtained from an individual mouse. Mean ± SEM are indicated. (A-C) *, p < 0.05; **, p < 0.01; ***, p < 0.001; ****, p < 0.0001 compared with control condition by one-way ANOVA followed by the Dunnet's post-test

To explore the mechanism underlying the arrest of T-cells in the colonic mucosa, we next performed transwell in vitro assays. Accordingly, we first assessed the migration of IEL towards different propionate concentrations. The results showed that propionate 100 μM – 300 μM induces a significant migration of TCRαβ^+^ T-cells (Fig. 7A). Since CXCL11-mediated stimulation of CXCR3 has been consistently involved in the lymphocyte infiltration into the CNS in mice and humans upon CNS autoimmunity [11, 43–47], we next addressed whether propionate-mediated GPR43 stimulation was able to affect the CXCL11-CXCR3 migration of IEL TCRαβ^+^ T-cells. For this purpose, we treated IEL with or without propionate for 1 h and then placed them on the top chamber to determine the extent of TCRαβ^+^ T-cells migration towards CXCL11 into the bottom transwell chamber. The results show that treating IEL TCRαβ^+^ T-cells with propionate attenuates their migration toward CXCL11 (Fig. 7B). Interestingly, this effect was selective for CD8^+^ T-cells but was not observed on CD4^+^ T-cells (Fig. 7C). Altogether these results suggest that propionate-mediated GPR43 stimulation on IEL TCRαβ T-cells retains these cells in the colonic epithelium and avoid the CXCL11-mediated recruitment of these lymphocytes into the CNS.

### GPR43 stimulation on IEL TCRαβ T-cells dampens gut inflammation

**Fig. 8:**
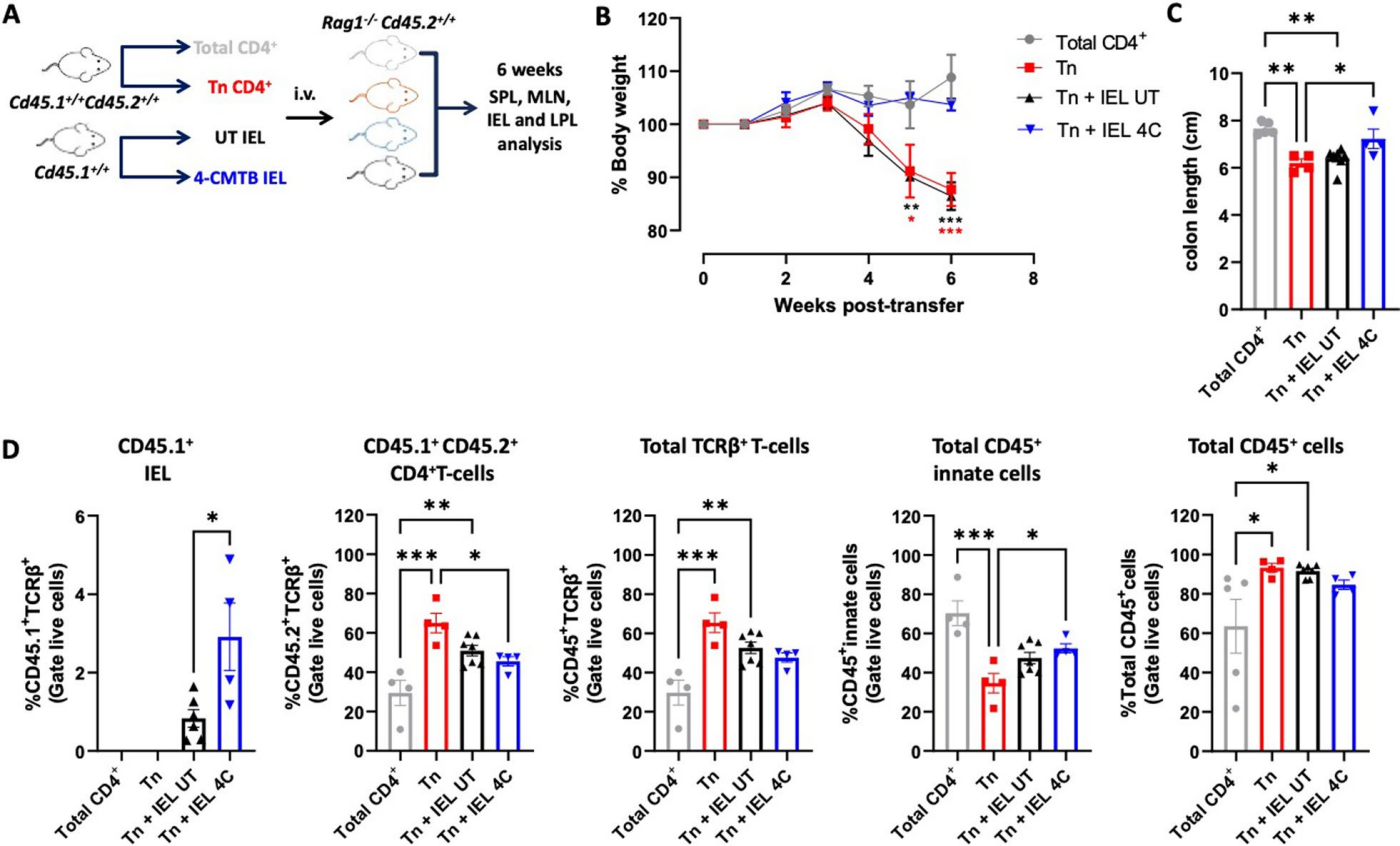
Pharmacologic stimulation of GPR43 on IEL induces an anti-inflammatory effect in experimental colitis. Inflammatory colitis was induced in *Rag1*^*−/−*^* Cd45.2*^+*/*+^ mice by the i.v. transference of 5 × 10^5^ naïve CD45.1^+^CD45.2^+^ CD4^+^ T-cells. In addition, some groups of mice received 5 × 10^5^ of IEL treated with 4-CMTB (blue) or untreated (UT black). As a control, another group received only total CD45.1^+^CD45.2^+^ CD4^+^ T-cells (grey). **A** Schematic illustration of the experimental design. **B** Body weight was weekly registered throughout the whole time and the percentage of body weight change relative to the initial weight was quantified. Values represent mean ± SEM. **C** At week 6, mice were sacrificed and the colon length was assessed. Each symbol represents data obtained from an individual mouse. **D** MNC were isolated from the colonic lamina propria 6 weeks after the transfer and the percentage of infiltration of each population was determined by flow cytometry: therapeutic IEL (CD45.1^+^), donor CD4^+^ T-cells (CD45.1^+^ CD45.2^+^), Total TCRβ^+^ T-cells (CD45.1^+^TCRβ^+^); Total innate CD45^+^ cells (CD45^+^TCRβ^−^) and total leukocytes (CD45^+^). **B**–**D** Mean ± SEM from 4 to 7 mice per group. *, p < 0.05; **, p < 0.01; ***, p < 0.001 by one-way ANOVA followed by Dunnett’s post-hoc test (**B**, comparing different experimental groups in each single day), or by Tukey’s post-hoc test (**C** and **D**). **B** Black asterisks correspond to comparisons between Tn + IEL UT and Total CD4^+^, whilst red asterisks correspond to comparisons between Tn and Total CD4^+^ groups

The results above indicate that GPR43 stimulation in IEL TCRαβ^+^ T-cells induces the retention of pro-inflammatory T-cells in the colonic mucosa and changes their cytokine expression to an anti-inflammatory profile, thus dampening CNS autoimmunity. To analyse whether the anti-inflammatory potential of GPR43 stimulation in IEL TCRαβ^+^ T-cells is extended to other inflammatory conditions outside the CNS, we next used a mouse model of inflammatory colitis. For this purpose, we transferred naïve CD4^+^ T-cells into lymphopenic mice, a situation that induces gut inflammation in the presence of commensal microbiota [52, 53]. Concomitantly, IEL isolated from WT mice were treated with a selective GPR43 agonist ex vivo and then transferred into experimental mice, and the disease severity was evaluated as the loss of body weight over time (Fig. 8A). The results show that mice receiving GPR43-stimulated IEL displayed a substantial reduction in body weight loss compared to those receiving untreated IEL or those receiving only naïve CD4^+^ T-cells in the absence of IEL transference (Fig. 8B). After six weeks of colitis induction, mice were sacrificed, and the colon shortening was evaluated as a macroscopic parameter of colon inflammation [52]. According to the effect observed in weight loss, mice receiving 4-CMTB-treated IEL did not present a significant colon shortening. Conversely, the mice receiving untreated IEL or the control group receiving only naïve CD4^+^ T-cells without IEL transference displayed a significant reduction in the colon length (Fig. 8C). These results indicate that GPR43 stimulation on IEL may also exert an anti-inflammatory effect in the colon, dampening the development of chronic inflammatory colitis. To analyse the mechanism underlying the anti-inflammatory effect of GPR43-stimulated IEL in this model of inflammatory colitis, we also determined the extent of infiltration of donor CD4^+^ T-cells (CD45.1^+^ CD45.2^+^), “therapeutic” IEL (CD45.1^+^), and total leukocyte (CD45^+^) in the colonic epithelium. According to the role of GPR43-signalling in the retention of TCRαβ^+^ T-cells in the colonic epithelium (Figs. 5 and 6), we observed that mice receiving 4-CMTB-treated IEL retained a higher extent of “therapeutic” donor IEL TCRαβ^+^ T-cells (CD45.1^+^) in the epithelium compared with those mice receiving untreated IEL (Fig. 8D). According to this, those mice receiving 4-CMTB-treated IEL but not those receiving untreated IEL presented a significant reduction in the extent of infiltration of inflammatory donor CD4^+^ T-cells (CD45.1^+^ CD45.2^+^; Fig. 8D). Similar results were obtained when the total number of TCRαβ^+^ T-cells were analysed in the colonic epithelium, including inflammatory donor CD4^+^ T-cells and “therapeutic” donor IEL (CD45^+^ TCRαβ^+^ T-cells; Fig. 8D). Moreover, despite the extent of total innate cells infiltrating the colonic epithelium was higher in those mice receiving 4-CMTB-treated IEL, the total leukocyte infiltration (CD45^+^ cells) in the colonic epithelium was increased only in those mice receiving untreated IEL, but not in those receiving 4-CMTB-treated IEL (Fig. 8D). Thus, these results suggest that GPR43-signalling favours the retention of anti-inflammatory IEL TCRαβ^+^ T-cells in the colonic epithelium, reducing the infiltration of inflammatory donor CD4^+^ T-cells and dampening the extent of total leukocyte infiltration in the colonic mucosa, alleviating inflammatory colitis.

## Discussion

Three non-excluding mechanisms have been proposed to explain how the gut microbiota might regulate CNS pathologies [54]: (1) Through the secretion of metabolites, neuropeptides, and neurotransmitters that directly stimulate their receptors on neurons of the enteric nervous system regulating neural signals that affect vagal transmission and thereby the CNS. (2) By producing metabolites that can diffuse through the intestinal wall, entering into the blood circulation, and acting in other organs, including the CNS. (3) Through the production of mediators that stimulate their receptors or enzymes expressed on immune cells, such as fatty acids and some neurotransmitters, thereby shaping the immune response. Our study here elucidates a mechanism of the third type. Our findings reveal a novel gut-brain axis mechanism by which bacterial-derived SCFAs secreted in the colonic mucosa might control the inflammatory/anti-inflammatory behaviour of mucosal T-cells and the CNS tropism of autoreactive lymphocytes. Furthermore, our study shows GPR43 expressed on colonic T-cells as a promising therapeutic target for CNS autoimmunity.

Other recent studies performed in animal models have shown how T-cells from the gut mucosa might be recruited into extraintestinal tissues under inflammatory conditions, including the recruitment into the CNS upon EAE [55] or stroke [56] and the lymphocytes infiltration into the retina upon experimental autoimmune uveitis (EAU) [57]. Interestingly, in the latter study, the oral administration of propionate suppressed the migration of intestinal T-cells into the spleen. Although the results were not statistically significant, that study also showed a trend of decreasing infiltration of intestinal leukocytes into the retina in those animals treated with propionate [57]. Thus, this latter study supports our findings suggesting that propionate-mediated GPR43 stimulation avoids lymphocyte emigration from the gastrointestinal mucosa. This mechanism reveals GPR43 expressed on colonic T-cells as a critical molecular target, which should be considered for designing therapeutic interventions for treating CNS autoimmunity. Our results encourage the development of strategies to exert the selective activity of GPR43-signalling in colonic T-cells employing viral vector-mediated approaches or by generating selective drugs.

Previous studies have provided pharmacologic and genetic evidence about the chemotactic potential of GPR43 stimulation in neutrophils. In this regard, it has been shown that mouse and human neutrophils migrate toward SCFAs in transwell assays [58–61]. Here we described how GPR43-signalling induced the selective chemotaxis of IEL TCRαβ^+^ T-cells into the colonic epithelium. Thus, these findings indicate that SCFAs produced by the commensal microbiota under homeostatic conditions represent a critical homing signal to retain these lymphocytes in the gut mucosa. According to the selective decrease of intestinal propionate levels and GPR43 down-regulation on IEL TCRαβ^+^ T-cells observed at the peak of EAE manifestation, these cells leave the colonic epithelium at this disease stage. These results suggest that propionate is especially important in the GPR43-mediated signalling associated with the TCRαβ^+^ T-cells retention in the colonic epithelium, rather than butyrate and acetate.

Considering that acetate:propionate:butyrate proportions in the colonic mucosa are 60:25:15 [62], and that GPR43 affinities are 52 μM, 31 μM, and 100 μM, respectively [63–65], it is expected a similar extent of GPR43 stimulation by acetate and propionate, which rule out the possibility that propionate stimulates colonic GPR43 more vigorously than acetate. A potential explanation for the particular relevance of propionate in the mechanism described here is that GPR43 expressed on IEL TCRαβ^+^ T-cells could form part of a heteromeric complex with other G protein-coupled receptors (GPCRs). In this regard, many studies have shown that GPCRs generally are assembled as homo- or hetero-oligomers [66]. Compared with GPCRs homomers, the assembly of GPCRs heteromers leads to changes in the identity and affinity for agonist recognition, in the signalling pathways coupled with, and in the trafficking of the individual protomers conforming the heteromer, thus strongly affecting their physiological function [67, 68]. Particularly GPR43 has been found to form heteromers with GPR41, a heteromeric complex coupled to intracellular signalling pathways different from that coupled to homomeric GPR43 [69]; however, the differences in affinities for SCFAs between homomeric GPR43 and the GPR43:GPR41 heteromer have not been yet explored. Thereby, it is tempting to speculate that IEL TCRαβ T-cells may express a GPR43:GPR41 heteromeric receptor with selective affinity for propionate; nevertheless, this possibility should be experimentally tested in future studies.

Interestingly, a recent work with human samples studied the association between the gut microbiome and migratory markers in peripheral blood lymphocytes obtained from MS patients and healthy controls. The authors found that MS was associated with reduced diversity in the gut microbiome and increased populations of CD4^+^ and CD8^+^ T-cells expressing the CNS-homing receptor CXCR3 [40]. Reduced diversity of the gut microbiome was characterised by an increased abundance in *Bacteroides* and reduced abundance in *Firmicutes* taxa [40]. Similarly, in the present study, we found a significant increase in the abundance of *Bacteroides* and a decreased abundance of *Firmicutes* in the gut microbiota at the peak of EAE manifestation, which was accompanied by a higher number of CXCR3^+^ TCRαβ^+^ T-cells infiltrating the CNS after the peak of the disease manifestation. Thus, our findings suggest that the microbiome-mediated regulation of lymphocytes associated with CNS autoimmunity seems similar in humans and mice. Moreover, a more detailed analysis of the relative abundance of bacteria belonging to the *Firmicutes* taxa revealed a sharp reduction of *Erysipelotrichaceae* at the peak of EAE manifestation, which has been strongly correlated with the production of propionic acid [70]. Therefore, our analysis of the gut microbiome provides a potential explanation of why the levels of propionate are decreased at the peak of EAE.

Previous works have shown anti-inflammatory effects mediated by GPR43-signalling in lymphocytes, including B cells [71] and Treg [22]. In this regard, Smith and colleagues demonstrated that, through the stimulation of GPR43, SCFAs regulate the size and function of the colonic Treg pool and protect against colitis [22]. Moreover, Yao and colleagues have recently shown that GPR43-signalling attenuates the development of collagen-induced arthritis in mice, which is dependent on B-cells [71]. Accordingly, the administration of SCFAs has been proven to ameliorate two different mouse models of lymphocyte-driven autoimmune disorders, including EAE and collagen-induced arthritis. This effect was accompanied by reduced Th1 response and increased Treg function [25]. Here, we described how GPR43 stimulation of colonic IEL also contributes to an anti-inflammatory effect by promoting a change in the profile of cytokines and surface molecules expressed by TCRαβ^+^ T-cells from pro-inflammatory to anti-inflammatory and by arresting autoreactive T-cells in the colonic mucosa. Of note, this anti-inflammatory effect mediated by GPR43 signalling on colonic IEL has an impact not only on CNS autoimmunity but also on inflammatory colitis. Thus, the present study contributes significantly to the growing knowledge of the complex regulation of the immune system by SCFAs and its impact on the development of autoimmune disorders.

A previous study indicated that GPR43-signalling promotes IL-10 and limits IFNγ production on T-cells specific for an antigen present in the gut microbiota in a model of inflammatory colitis [72]. Similarly, we show that SCFAs reduced IFNγ production and induced the production of the anti-inflammatory cytokine IL-10 by colonic IEL TCRαβ T-cells (Fig. 3). Our results obtained here using in vivo and ex vivo approaches indicate that propionate directly affects IEL TCRαβ T-cells, promoting an anti-inflammatory profile (Figs. 3C, 3E, and Additional file 1: Fig. S3C) and favouring the colonic tropism (Fig. 7). Conversely, the results show that the ex vivo treatment of LPL with propionate did not affect PD-1 and IL-10 expression by TCRαβ T-cells (Fig. 3D). In contrast, the oral administration of propionate induced a higher frequency of IL-10^+^ and PD-1^+^ TCRαβ^+^ T-cells in the colonic lamina propria (Additional file 1: Fig. S3C). Together, these results suggest that in vivo, propionate act on other sources of cells, indirectly promoting an anti-inflammatory profile on the LPL TCRαβ T-cells. In this regard, other cells expressing SCFAs receptors in the colonic mucosa, such as DCs, macrophages, innate lymphoid cells, or epithelial cells [23, 73–75], could be responsible for this indirect effect on the LPL TCRαβ T-cells. Future studies should be carried out to address this exciting issue.

## Conclusions

Our findings suggest a homeostatic mechanism by which SCFAs secreted in the colonic mucosa by the commensal microbiota under healthy conditions would stimulate GPR43 on mucosal TCRαβ^+^ T-cells inducing an anti-inflammatory functional profile and their retention in this tissue. Nonetheless, CNS autoimmunity-associated microbial dysbiosis would result in a selective reduction in the propionate-mediated GPR43 stimulation in mucosal lymphocytes, consequently inducing a switch of the functional phenotype of colonic T-cells from anti-inflammatory to pro-inflammatory and promoting the scape of these T-cells from the colonic mucosa to reach the CNS.

## Supplementary Information

**Additional file 1: Figure S1**. Intestinal microbiome composition changes during EAE. Stool samples were collected from healthy miceand EAE mice at indicated time points and microbial composition was determined.. Alpha diversity metrics. Evaluation of microbial richness and diversity within each group was assessed using the *Chao1* index and the Shannon Index.Analysis of beta diversity.Values represent the divergence of intestinal microbiome throughout EAE progression.PCoA analysis based on unweighted UniFrac throughout EAE progression.Intestinal microbiome composition at the Phylum level was analysed by mean percent ASV abundance and represented as pie chart for each time-point.Comparison between *Bacteroidota* and *Firmicutes* Phyla abundance throughout EAE progression.Intestinal microbiome composition at Family level of top three more abundant families was analysed by mean percent ASV abundance.In the box plots, error bars correspond to minimum and maximum data points, the horizontal line corresponds to the median, and the box contains 75% of the data points. Data were obtained from 4–11 mice per group. *, p < 0.05; **, p < 0.01 by one-way ANOVA followed by Tukey’s post-hoc test. **Figure S2**. GPR43 expression on IEL TCRαβ^+^ T-cells is upregulated by SCFAs.WT mice were non-treatedor treated with 250 mM SCFAs in the drinking water for two weeks and GPR43 expression was analysed in IEL CD4, CD8αβ and CD8αα subsets or total TCRαβ^+^ T-cells by flow cytometry. Scheme illustrating the experimental design.Quantification of GPR43 expression. Values are the MFI associated to GPR43 immunostaining.GPR43 expression was analysed in IEL CD4, CD8αβ and CD8αα subsets or total TCRαβ^+^ T-cells isolated from healthyor EAE mice at 25 dpi.Quantification of the clinical score throughout EAE development. The arrow indicates the time-point in which IEL were isolated.Quantification of GPR43 expression. Values are the MFI associated to GPR43 immunostaining.Representative histogram of GPR43 expression analysed by flow cytometry. Control, healthy miceand EAE mice.Each symbol represents data obtained from an individual mouse.Mean ± SD from 3–5 mice per group. *, p < 0.05 by one-way ANOVA followed by Dunnett’s post-hoc testor unpaired Student’s t-test. **Figure S3**. Oral administration of SCFAs reduces EAE manifestation by increasing IL-10 and PD-1 expression on colonic lymphocytes. WT mice were treated with 250 mM SCFAs in the drinking water and two weeks later EAE was induced. SCFAs treatment was extended until 25 dpi. Treatments with acetate, propionate, butyrateor only waterare indicated.Disease severity was evaluated over time. Values represent mean ± SEM from a representativeof three independent experiments.The average water consumption per mouse was determined along EAE development. Data were obtained from four mice per group. Each symbol represents data obtained from an individual mouse per day. At 25 dpi IEL and LPL were isolated, re-stimulated wih PMA/Iono/BFA and then the absolute numbers of TCRαβ^+^ subsets, and the production of IL-10 and expression of PD-1 by CD4, CD8αβ and CD8αα TCRαβ^+^ T-cells was quantified. Data were obtained from 3–6 mice per group. Each symbol represents data obtained from an individual mouse. Mean ± SEM are indicated.*, p < 0.05, **, p < 0.01; ***, p < 0.001; ****, p < 0.0001 by one-way ANOVA followed by Dunnett’s post-hoc test. Purple asterisks correspond to comparisons between control and butyrate groups, whilst green asterisks correspond to comparisons between control and propionate groups and red asterisk correspond to comparisons between control and acetate groups. **Figure S4**. Donor colonic IEL are preferentially retained in the colonic epithelium of recipients. IEL were isolated from *Cd45.1*^+*/*+^ congenic mice and then i.v. transferred into *Rag1*^*−/−*^* Cd45.2*^+*/*+^recipient mice. Spleen, mesenteric lymph nodes, colonic intraepithelial lymphocytesand colonic lamina propria lymphocytes were isolated from recipients at different time points after the transfer and the percentage of total donor cellsand donor T-cells were quantified by flow cytometry. Schematic illustration of the experimental design. Frequency of CD45.1^+^ and TCRβ^+^ donor cells distributed in the different tissues analysed. Values represent mean ± SEM from 3 mice per group. Representative dot plots of CD45.1^+^and CD45.2^+^cells and TCRαβ^+^ and TCRγδ^+^ sub-populations of donor CD45.1^+^ cells isolated from the colonic intraepithelial compartment.*, p < 0.05; **, p < 0.01; ***, p < 0.001; ****, p < 0.0001 comparing with values obtained at time cero for each particular experimental group by one-way ANOVA followed by Dunnett’s post-hoc test. The asterisk colours indicate the experimental group with which they are associated. **Figure S5**. Dynamics of lymphocytes in the colonic lamina propria during EAE development. Colonic LPL were isolated from EAE mice at indicated time points and the frequency of CD19^+^ B-cells, TCRγδ^+^ and total TCRαβ^+^ T-cells was analysed.The absolute number of total or CXCR3^+^cells from the CD4^+^, CD8αα^+^ and CD8αβ^+^ TCRαβ subsets of colonic LPL isolated from EAE mice at indicated time points was determined. Percentage of total CD4^+^, CD8αα^+^ and CD8αβ^+^ TCRαβ T-cells from LPL or those expressing CD103, α4β7 and CCR9 were determined at different time points during EAE development.Values represent the mean ± SEM from 3–6 mice per group. *, p < 0.05; **, p < 0.01 by One-way ANOVA followed by Dunnett’s post-hoc test comparing frequencies of each cell type at different time points with the control condition.

## Data Availability

The datasets used and/or analysed during the current study are available from the corresponding author on reasonable request.

## Abbreviations

ACK: Ammonium-chloride-potassium
Ab: Antibody
ABX: Antibiotics
CNS: Central nervous system
CD*n*: Cluster of differentiation *n*
EAE: Experimental autoimmune encephalomyelitis
dpi: Days post-induction
IEL: Intraepithelial lymphocytes
LPL: Lamina *propria* lymphocytes
MFI: Mean fluorescence intensity
MLN: Mesenteric lymph node
MOG: Myelin oligodendrocyte glycoprotein
MS: Multiple sclerosis
PD-1: Programmed cell death-1
pMOG: Peptide MOG_35-55_
PBMC: Peripheral blood mononuclear cells
Rag1: Recombination activating gene 1
Treg: Regulatory T-cells
SCFAs: Short-chain fatty acids
SPL: Spleen
Tbet: T-box transcription factor encoded by TBX21
Th*n*: T helper *n*

## References

[1] Lyte M (2013). Microbial endocrinology in the microbiome-gut-brain axis: how bacterial production and utilization of neurochemicals influence behavior. PLoS Pathog.

[2] Cox LM, Weiner HL (2018). Microbiota signaling pathways that influence neurologic disease. Neurotherapeutics J Am Soc Exp Neurother.

[3] Cani PD (2018). Human gut microbiome: hopes, threats and promises. Gut.

[4] Stefano GB, Pilonis N, Ptacek R, Raboch J, Vnukova M, Kream RM (2018). Gut, microbiome, and brain regulatory axis: relevance to neurodegenerative and psychiatric disorders. Cell Mol Neurobiol..

[5] Chu F, Shi M, Lang Y, Shen D, Jin T, Zhu J (2018). Gut microbiota in multiple sclerosis and experimental autoimmune encephalomyelitis: current applications and future perspectives. Mediators Inflamm.

[6] Pacheco R (2019). Cross-talk between T-cells and gut-microbiota in neurodegenerative disorders. Neural Regen Res.

[7] Goverman J (2009). Autoimmune T cell responses in the central nervous system. Nat Rev Immunol.

[8] Absinta M, Maric D, Gharagozloo M, Garton T, Smith MD, Jin J (2021). A lymphocyte-microglia-astrocyte axis in chronic active multiple sclerosis. Nature.

[9] Rothhammer V, Borucki DM, Tjon EC, Takenaka MC, Chao CC, Ardura-Fabregat A (2018). Microglial control of astrocytes in response to microbial metabolites. Nature.

[10] Yong VW (2022). Microglia in multiple sclerosis: protectors turn destroyers. Neuron.

[11] Prado C, Osorio-Barrios F, Falcon P, Espinoza A, Saez JJ, Yuseff MI (2021). Dopaminergic stimulation leads B-cell infiltration into the central nervous system upon autoimmunity. J Neuroinflamm.

[12] Kaushik DK, Bhattacharya A, Lozinski BM, Wee Yong V (2021). Pericytes as mediators of infiltration of macrophages in multiple sclerosis. J Neuroinflamm.

[13] Mcginley AM, Sutton CE, Edwards SC, Leane CM, Decourcey J, Teijeiro A (2020). Interleukin-17a serves a priming role in autoimmunity by recruiting Il-1beta-producing myeloid cells that promote pathogenic T cells. Immunity.

[14] Berer K, Gerdes LA, Cekanaviciute E, Jia X, Xiao L, Xia Z (2017). Gut microbiota from multiple sclerosis patients enables spontaneous autoimmune encephalomyelitis in mice. Proc Natl Acad Sci U S A.

[15] Lee YK, Menezes JS, Umesaki Y, Mazmanian SK (2011). Proinflammatory T-cell responses to gut microbiota promote experimental autoimmune encephalomyelitis. Proc Natl Acad Sci U S A.

[16] Koh A, De Vadder F, Kovatcheva-Datchary P, Backhed F (2016). From dietary fiber to host physiology: short-chain fatty acids as key bacterial metabolites. Cell.

[17] Waldecker M, Kautenburger T, Daumann H, Busch C, Schrenk D (2008). Inhibition of histone-deacetylase activity by short-chain fatty acids and some polyphenol metabolites formed in the colon. J Nutr Biochem.

[18] Arpaia N, Campbell C, Fan X, Dikiy S, Van Der Veeken J, Deroos P (2013). Metabolites produced by commensal bacteria promote peripheral regulatory T-cell generation. Nature.

[19] Zhou D, Pan Q, Liu XL, Yang RX, Chen YW, Liu C (2017). Clostridium Butyricum B1 alleviates high-fat diet-induced steatohepatitis in mice via enterohepatic immunoregulation. J Gastroenterol Hepatol.

[20] Fang L, Pang Z, Shu W, Wu W, Sun M, Cong Y (2018). Anti-Tnf therapy induces Cd4+ T-cell production of Il-22 and promotes epithelial repairs in patients with Crohn's disease. Inflamm Bowel Dis.

[21] Trompette A, Gollwitzer ES, Yadava K, Sichelstiel AK, Sprenger N, Ngom-Bru C (2014). Gut microbiota metabolism of dietary fiber influences allergic airway disease and hematopoiesis. Nat Med.

[22] Smith PM, Howitt MR, Panikov N, Michaud M, Gallini CA, Bohlooly YM (2013). The microbial metabolites, short-chain fatty acids, regulate colonic Treg cell homeostasis. Science.

[23] Singh N, Gurav A, Sivaprakasam S, Brady E, Padia R, Shi H, et al. Activation of Gpr109a, receptor for niacin and the commensal metabolite butyrate, suppresses colonic inflammation and carcinogenesis. Immunity. 2014;40:128–39. 10.1016/j.immuni.2013.12.007 PMC4305274 24412617

[24] Miyake S, Kim S, Suda W, Oshima K, Nakamura M, Matsuoka T (2015). Dysbiosis in the gut microbiota of patients with multiple sclerosis, with a striking depletion of species belonging to clostridia Xiva and Iv clusters. PLoS ONE.

[25] Mizuno M, Noto D, Kaga N, Chiba A, Miyake S (2017). The dual role of short fatty acid chains in the pathogenesis of autoimmune disease models. PLoS ONE.

[26] Haghikia A, Jorg S, Duscha A, Berg J, Manzel A, Waschbisch A (2015). Dietary fatty acids directly impact central nervous system autoimmunity via the small intestine. Immunity.

[27] Park J, Wang Q, Wu Q, Mao-Draayer Y, Kim CH (2019). Bidirectional regulatory potentials of short-chain fatty acids and their G-protein-coupled receptors in autoimmune neuroinflammation. Sci Rep.

[28] Reissig S, Hackenbruch C, Hovelmeyer N (2014). Isolation of T cells from the gut. Methods Mol Biol.

[29] Zabel BA, Agace WW, Campbell JJ, Heath HM, Parent D, Roberts AI (1999). Human G protein-coupled receptor Gpr-9-6/Cc chemokine receptor 9 is selectively expressed on intestinal homing T lymphocytes, mucosal lymphocytes, and thymocytes and is required for thymus-expressed chemokine-mediated chemotaxis. J Exp Med.

[30] Kunkel EJ, Campbell JJ, Haraldsen G, Pan J, Boisvert J, Roberts AI (2000). Lymphocyte Cc chemokine receptor 9 and epithelial thymus-expressed chemokine (Teck) expression distinguish the small intestinal immune compartment: epithelial expression of tissue-specific chemokines as an organizing principle in regional immunity. J Exp Med.

[31] Callahan BJ, Mcmurdie PJ, Rosen MJ, Han AW, Johnson AJ, Holmes SP (2016). Dada2: high-resolution sample inference from illumina amplicon data. Nat Methods.

[32] Quast C, Pruesse E, Yilmaz P, Gerken J, Schweer T, Yarza P (2013). The silva ribosomal RNA gene database project: improved data processing and web-based tools. Nucleic Acids Res.

[33] Mcmurdie PJ, Holmes S (2013). Phyloseq: an R package for reproducible interactive analysis and graphics of microbiome census data. PLoS ONE.

[34] Love MI, Huber W, Anders S (2014). Moderated estimation of fold change and dispersion for Rna-Seq data with Deseq2. Genome Biol.

[35] Berer K, Mues M, Koutrolos M, Rasbi ZA, Boziki M, Johner C (2011). Commensal microbiota and myelin autoantigen cooperate to trigger autoimmune demyelination. Nature.

[36] Yokote H, Miyake S, Croxford JL, Oki S, Mizusawa H, Yamamura T (2008). Nkt cell-dependent amelioration of a mouse model of multiple sclerosis by altering gut flora. Am J Pathol.

[37] Beura LK, Hamilton SE, Bi K, Schenkel JM, Odumade OA, Casey KA (2016). Normalizing the environment recapitulates adult human immune traits in laboratory mice. Nature.

[38] Ivanov II, Atarashi K, Manel N, Brodie EL, Shima T, Karaoz U (2009). Induction of intestinal Th17 cells by segmented filamentous bacteria. Cell.

[39] Gandy KAO, Zhang J, Nagarkatti P, Nagarkatti M (2019). The role of gut microbiota in shaping the relapse-remitting and chronic-progressive forms of multiple sclerosis in mouse models. Sci Rep.

[40] Choileain SN, Kleinewietfeld M, Raddassi K, Hafler DA, Ruff WE, Longbrake EE (2020). Cxcr3+ t cells in multiple sclerosis correlate with reduced diversity of the gut microbiome. J Transl Autoimmun.

[41] Ochoa-Reparaz J, Mielcarz DW, Ditrio LE, Burroughs AR, Foureau DM, Haque-Begum S (2009). Role of gut commensal microflora in the development of experimental autoimmune encephalomyelitis. J Immunol.

[42] Lavasani S, Dzhambazov B, Nouri M, Fak F, Buske S, Molin G (2010). A novel probiotic mixture exerts a therapeutic effect on experimental autoimmune encephalomyelitis mediated by Il-10 producing regulatory T cells. PLoS ONE.

[43] Saraste M, Penttila TL, Airas L (2016). Natalizumab treatment leads to an increase in circulating Cxcr3-expressing B cells. Neurol Neuroimmunol Neuroinflamm.

[44] Van Langelaar J, Rijvers L, Janssen M, Wierenga-Wolf AF, Melief MJ, Siepman TA (2019). Induction of brain-infiltrating T-Bet-expressing B cells in multiple sclerosis. Ann Neurol.

[45] Sorensen TL, Trebst C, Kivisakk P, Klaege KL, Majmudar A, Ravid R (2002). Multiple sclerosis: a study of Cxcl10 and Cxcr3 Co-localization in the inflamed central nervous system. J Neuroimmunol.

[46] Wang W, Chong WP, Li C, Chen Z, Wu S, Zhou H (2019). Type I interferon therapy limits CNS autoimmunity by inhibiting Cxcr3-mediated trafficking of pathogenic effector T cells. Cell Rep.

[47] Zhou YQ, Liu DQ, Chen SP, Sun J, Zhou XR, Xing C (2019). The role of Cxcr3 in neurological diseases. Curr Neuropharmacol.

[48] Cassani B, Villablanca EJ, Quintana FJ, Love PE, Lacy-Hulbert A, Blaner WS (2011). Gut-tropic T cells that express integrin Alpha4beta7 and Ccr9 are required for induction of oral immune tolerance in mice. Gastroenterology.

[49] Schon MP, Arya A, Murphy EA, Adams CM, Strauch UG, Agace WW (1999). Mucosal T lymphocyte numbers are selectively reduced in integrin Alpha E (Cd103)-deficient mice. J Immunol.

[50] Masopust D, Schenkel JM (2013). The integration of T cell migration, differentiation and function. Nat Rev Immunol.

[51] Bettelli E, Pagany M, Weiner HL, Linington C, Sobel RA, Kuchroo VK (2003). Myelin oligodendrocyte glycoprotein-specific T cell receptor transgenic mice develop spontaneous autoimmune optic neuritis. J Exp Med.

[52] Ostanin DV, Bao J, Koboziev I, Gray L, Robinson-Jackson SA, Kosloski-Davidson M (2009). T cell transfer model of chronic colitis: concepts, considerations, and tricks of the trade. Am J Physiol Gastrointest Liver Physiol.

[53] Contreras F, Prado C, Gonzalez H, Franz D, Osorio-Barrios F, Osorio F (2016). Dopamine receptor D3 signaling on Cd4+ T cells favors Th1- and Th17-mediated immunity. J Immunol.

[54] Campos-Acuna J, Elgueta D, Pacheco R (2019). T-cell-driven inflammation as a mediator of the gut-brain axis involved in Parkinson's disease. Front Immunol.

[55] Hiltensperger M, Beltran E, Kant R, Tyystjarvi S, Lepennetier G, Dominguez Moreno H (2021). Skin and gut imprinted helper T cell subsets exhibit distinct functional phenotypes in central nervous system autoimmunity. Nat Immunol.

[56] Brea D, Poon C, Benakis C, Lubitz G, Murphy M, Iadecola C (2021). Stroke affects intestinal immune cell trafficking to the central nervous system. Brain Behav Immunity.

[57] Nakamura YK, Janowitz C, Metea C, Asquith M, Karstens L, Rosenbaum JT (2017). Short chain fatty acids ameliorate immune-mediated uveitis partially by altering migration of lymphocytes from the intestine. Sci Rep.

[58] Dahlstrand Rudin A, Khamzeh A, Venkatakrishnan V, Basic A, Christenson K, Bylund J (2021). Short chain fatty acids released by fusobacterium nucleatum are neutrophil chemoattractants acting via free fatty acid receptor 2 (Ffar2). Cell Microbiol.

[59] Vinolo MA, Ferguson GJ, Kulkarni S, Damoulakis G, Anderson K, Bohlooly YM (2011). Scfas induce mouse neutrophil chemotaxis through the Gpr43 receptor. PLoS ONE.

[60] Frei R, Nordlohne J, Huser U, Hild S, Schmidt J, Eitner F (2021). Allosteric targeting of the Ffa2 receptor (Gpr43) restores responsiveness of desensitized human neutrophils. J Leukoc Biol.

[61] Sina C, Gavrilova O, Forster M, Till A, Derer S, Hildebrand F (2009). G protein-coupled receptor 43 is essential for neutrophil recruitment during intestinal inflammation. J Immunol.

[62] Marques FZ, Mackay CR, Kaye DM (2018). Beyond gut feelings: how the gut microbiota regulates blood pressure. Nat Rev Cardiol.

[63] Brown AJ, Goldsworthy SM, Barnes AA, Eilert MM, Tcheang L, Daniels D (2003). The orphan G protein-coupled receptors Gpr41 and Gpr43 are activated by propionate and other short chain carboxylic acids. J Biol Chem.

[64] Le Poul E, Loison C, Struyf S, Springael JY, Lannoy V, Decobecq ME (2003). Functional characterization of human receptors for short chain fatty acids and their role in polymorphonuclear cell activation. J Biol Chem.

[65] Nilsson NE, Kotarsky K, Owman C, Olde B (2003). Identification of a free fatty acid receptor, Ffa2r, expressed on leukocytes and activated by short-chain fatty acids. Biochem Biophys Res Commun.

[66] Ng HK, Chow BK (2015). Oligomerization of family B Gpcrs: exploration in inter-family oligomer formation. Front Endocrinol.

[67] Fuxe K, Borroto-Escuela DO, Marcellino D, Romero-Fernandez W, Frankowska M, Guidolin D (2012). Gpcr heteromers and their allosteric receptor–receptor interactions. Curr Med Chem.

[68] Borroto-Escuela DO, Tarakanov AO, Guidolin D, Ciruela F, Agnati LF, Fuxe K (2011). Moonlighting characteristics of G protein-coupled receptors: focus on receptor heteromers and relevance for neurodegeneration. IUBMB Life.

[69] Ang Z, Xiong D, Wu M, Ding JL (2018). Ffar2-Ffar3 receptor heteromerization modulates short-chain fatty acid sensing. FASEB J.

[70] Li LL, Wang YT, Zhu LM, Liu ZY, Ye CQ, Qin S (2020). Inulin with different degrees of polymerization protects against diet-induced endotoxemia and inflammation in association with gut microbiota regulation in mice. Sci Rep.

[71] Yao Y, Cai X, Zheng Y, Zhang M, Fei W, Sun D (2022). Short-chain fatty acids regulate B cells differentiation via the Ffa2 receptor to alleviate rheumatoid arthritis. Br J Pharmacol.

[72] Sun M, Wu W, Chen L, Yang W, Huang X, Ma C (2018). Microbiota-derived short-chain fatty acids promote Th1 cell Il-10 production to maintain intestinal homeostasis. Nat Commun.

[73] Sepahi A, Liu Q, Friesen L, Kim CH (2021). Dietary fiber metabolites regulate innate lymphoid cell responses. Mucosal Immunol.

[74] Lavoie S, Chun E, Bae S, Brennan CA, Gallini Comeau CA, Lang JK (2020). Expression of free fatty acid receptor 2 by dendritic cells prevents their expression of interleukin 27 and is required for maintenance of mucosal barrier and immune response against colorectal tumors in mice. Gastroenterology.

[75] Bilotta AJ, Ma C, Yang W, Yu Y, Yu Y, Zhao X (2021). Propionate enhances cell speed and persistence to promote intestinal epithelial turnover and repair. Cell Mol Gastroenterol Hepatol.

