# Supplementary figures and images for: GPR43 stimulation on TCRαβ+ intraepithelial colonic lymphocytes inhibits the recruitment of encephalitogenic T-cells into the central nervous system and attenuates the development of autoimmunity
Source: J Neuroinflammation. 2023 Jun 1;20:135. doi: 10.1186/s12974-023-02815-9 (PMC10233874; doi:10.1186/s12974-023-02815-9)

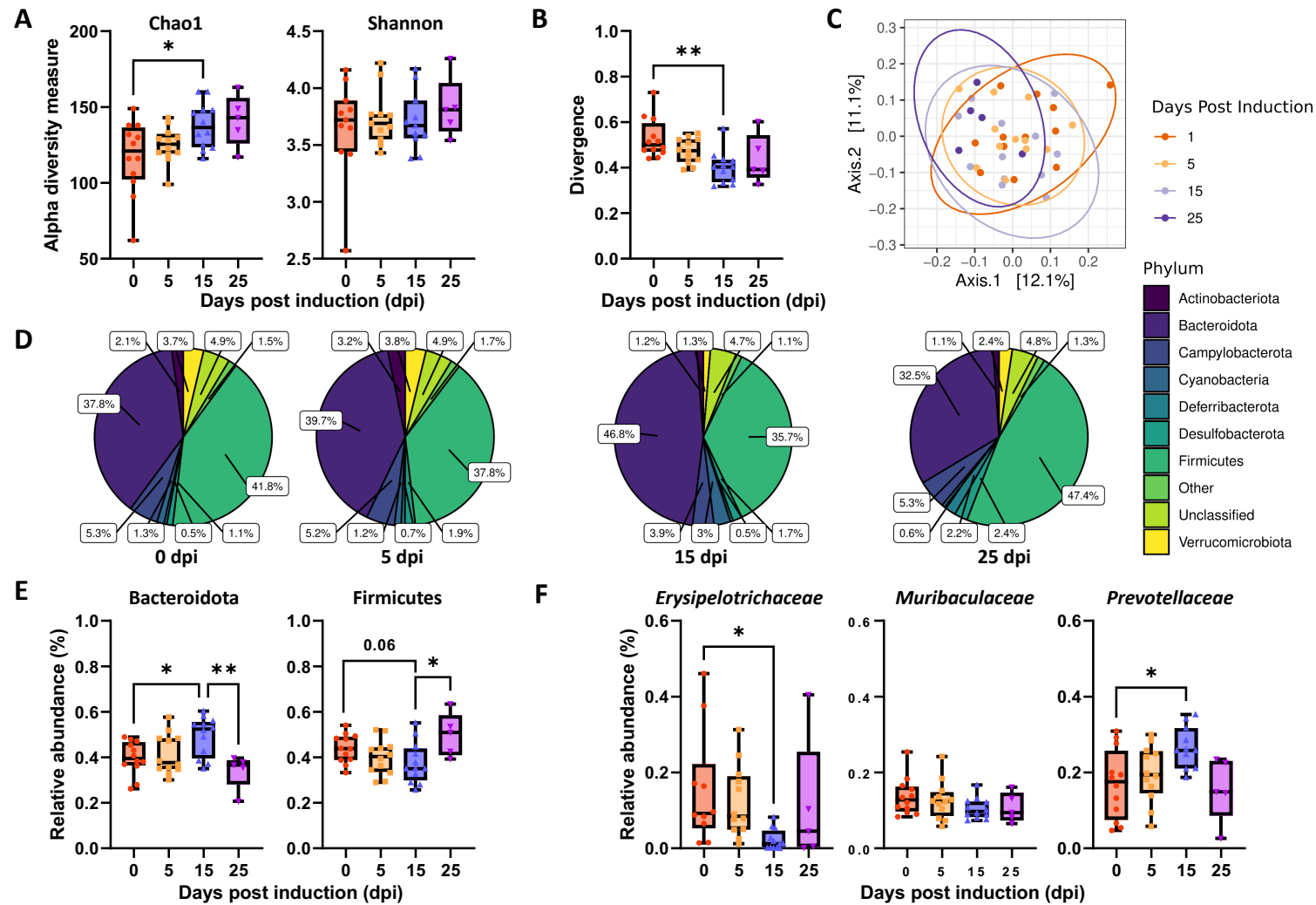

Figure S1

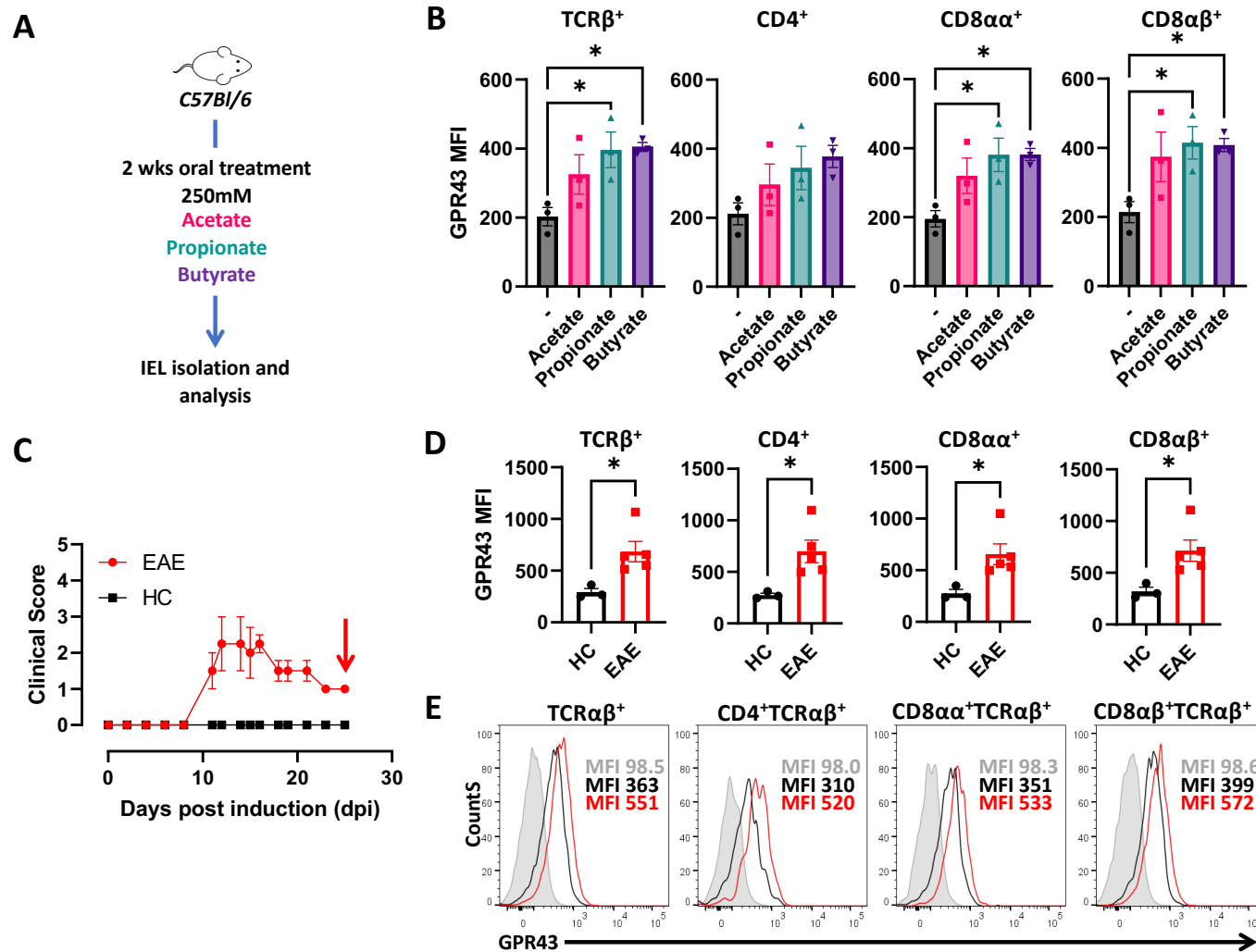

Figure S2

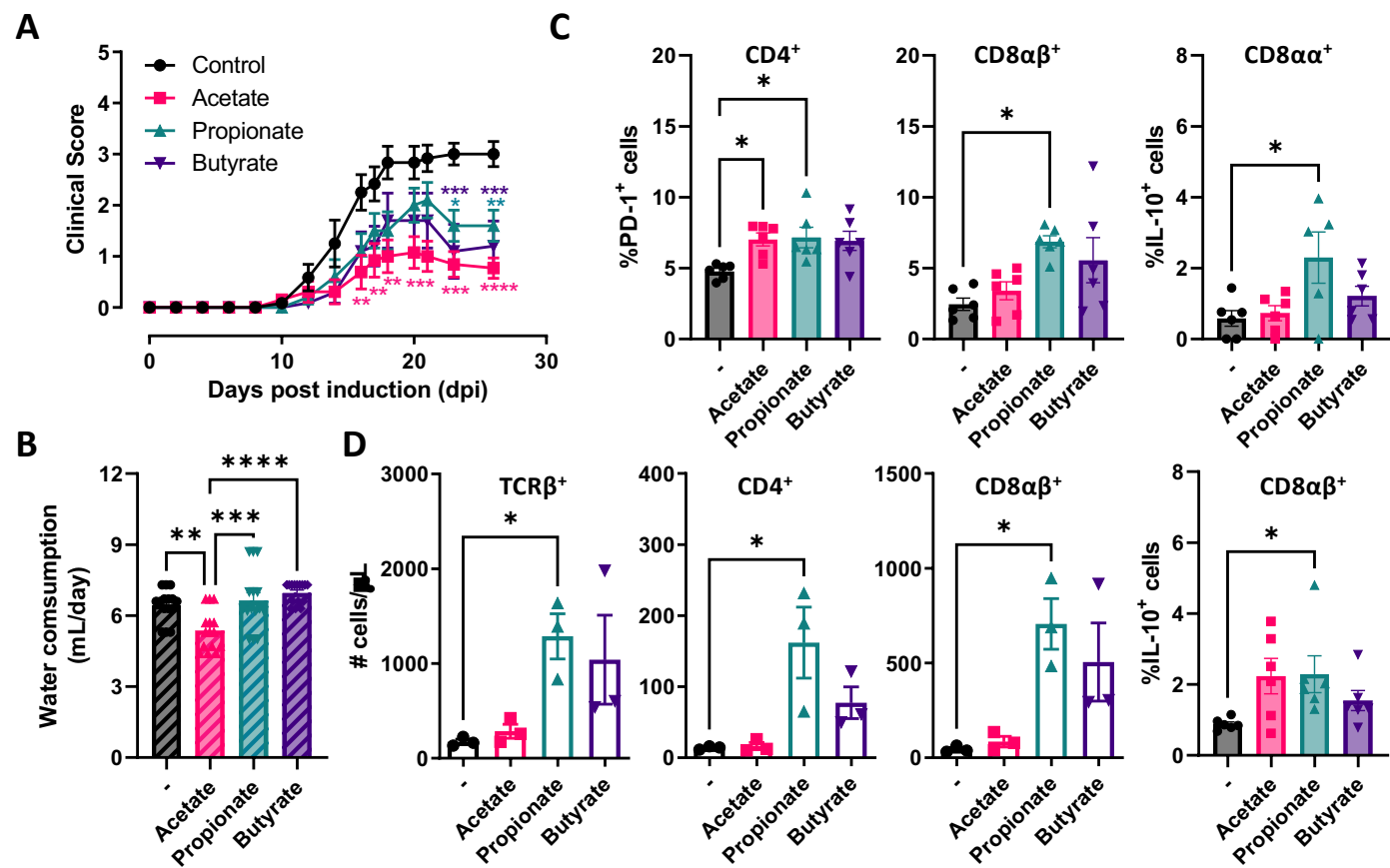

Figure S3

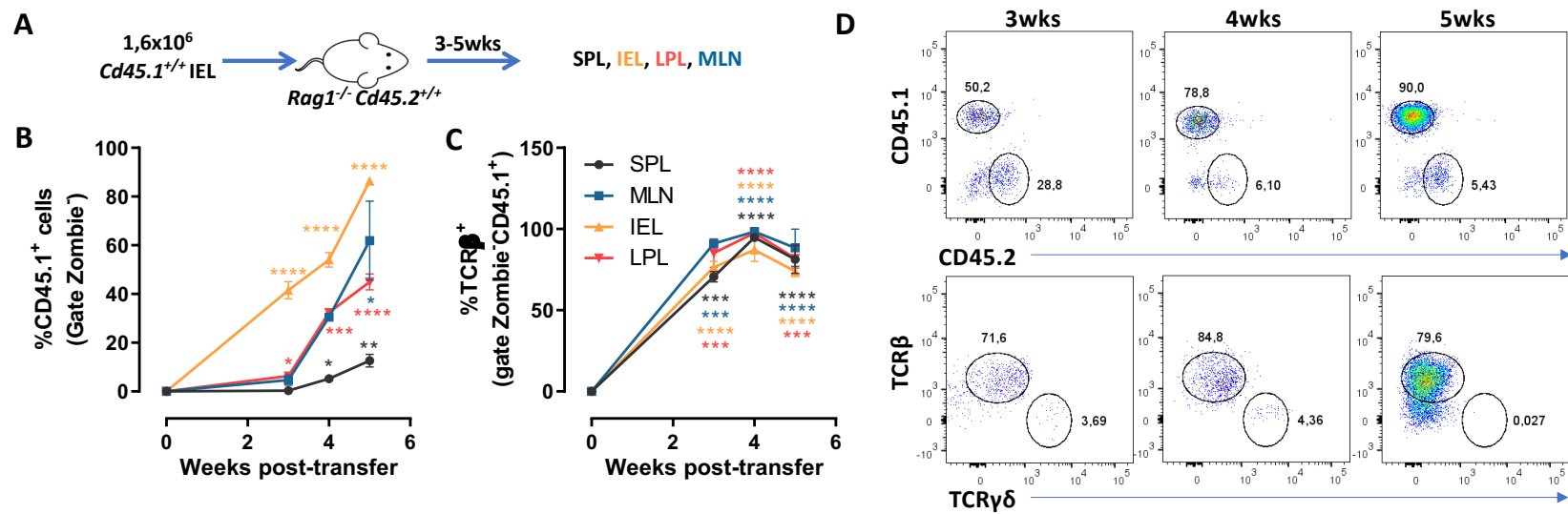

Figure S4

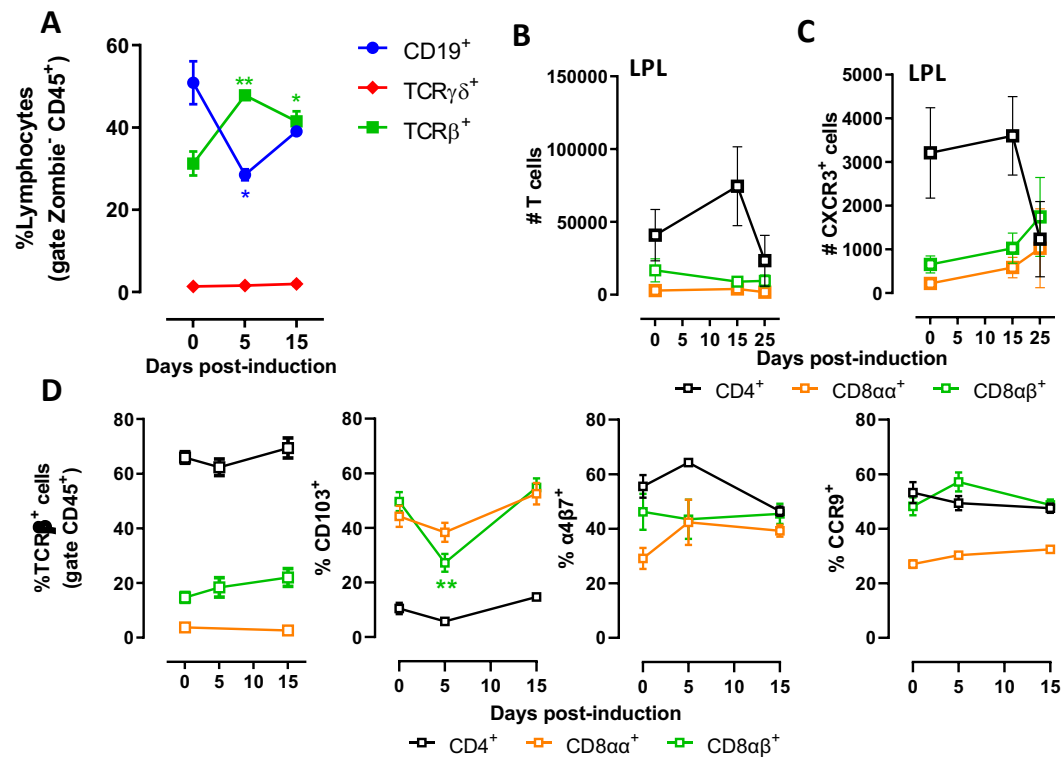

Figure S5

Supplement: Supplementary file 1 — Additional file 1: Figure S1. Intestinal microbiome composition changes during EAE. Stool samples were collected from healthy miceand EAE mice at indicated time points and microbial composition was determined.. Alpha diversity metrics. Evaluation of microbial richness and diversity within each group was assessed using the Chao1 index and the Shannon Index.Analysis of beta diversity.Values represent the divergence of intestinal microbiome throughout EAE progression.PCoA analysis based on unweighted UniFrac throughout EAE progression.Intestinal microbiome composition at the Phylum level was analysed by mean percent ASV abundance and represented as pie chart for each time-point.Comparison between Bacteroidota and Firmicutes Phyla abundance throughout EAE progression.Intestinal microbiome composition at Family level of top three more abundant families was analysed by mean percent ASV abundance.In the box plots, error bars correspond to minimum and maximum data points, the horizontal line corresponds to the median, and the box contains 75% of the data points. Data were obtained from 4–11 mice per group. *, p < 0.05; **, p < 0.01 by one-way ANOVA followed by Tukey’s post-hoc test. Figure S2. GPR43 expression on IEL TCRαβ+ T-cells is upregulated by SCFAs.WT mice were non-treatedor treated with 250 mM SCFAs in the drinking water for two weeks and GPR43 expression was analysed in IEL CD4, CD8αβ and CD8αα subsets or total TCRαβ+ T-cells by flow cytometry. Scheme illustrating the experimental design.Quantification of GPR43 expression. Values are the MFI associated to GPR43 immunostaining.GPR43 expression was analysed in IEL CD4, CD8αβ and CD8αα subsets or total TCRαβ+ T-cells isolated from healthyor EAE mice at 25 dpi.Quantification of the clinical score throughout EAE development. The arrow indicates the time-point in which IEL were isolated.Quantification of GPR43 expression. Values are the MFI associated to GPR43 immunostaining.Representative histogram of GPR43 expression a [file 12974_2023_2815_MOESM1_ESM.pdf]
